# Trichostatin A ameliorates renal tubulointerstitial fibrosis through modulation of the JNK-dependent Notch-2 signaling pathway

**DOI:** 10.1038/s41598-017-15162-6

**Published:** 2017-11-03

**Authors:** Chun-Wu Tung, Yung-Chien Hsu, Chang-Jhih Cai, Ya-Hsueh Shih, Ching-Jen Wang, Pey-Jium Chang, Chun-Liang Lin

**Affiliations:** 1 Department of Nephrology, Chang Gung Memorial Hospital, Chiayi, Taiwan; 2 College of Medicine, Chang Gung University, Taoyuan, Taiwan; 3 Graduate Institute of Clinical Medical Sciences, Chang Gung University, Taoyuan, Taiwan; 4 Kidney and Diabetic Complications Research Team (KDCRT), Chang Gung Memorial Hospital, Chiayi, Taiwan; 5 Kidney Research Center, Chang Gung Memorial Hospital, Taipei, Taiwan; 6 Center for Shockwave Medicine and Tissue Engineering, Kaohsiung Chang Gung Memorial Hospital and Chang Gung University College of Medicine, Kaohsiung, Taiwan; 7 Department of Orthopedic Surgery, Chang Gung Memorial Hospital, Kaohsiung, Taiwan

## Abstract

Renal fibrosis is the final common pathological feature in a variety of chronic kidney disease. Trichostatin A (TSA), a histone deacetylase inhibitor, reportedly attenuates renal fibrosis in various kidney disease models. However, the detailed molecular action of TSA in ameliorating renal fibrotic injury is not yet fully understood. In a cultured renal fibroblastic cell model, we showed that TGF-β1 triggers upregulation of α-SMA and fibronectin, two hallmarks of myofibroblastic activation. During the course of TGF-β1 treatment, activation of Smad2/3, p38, ERK, JNK and Notch-2 was also detected. Under the conditions, administration of TSA significantly decreased TGF-β1-stimulated expression of α-SMA, fibronectin, phospho-JNK, and cleaved Notch-2; however, the levels of phospho-Smad2/3, phospho-p38 and phospho-ERK remained unchanged. Pharmacological inhibition of different signaling pathways and genetic knockdown of Notch-2 further revealed JNK as an upstream effector of Notch-2 in TGF-β1-mediated renal fibrosis. Consistently, we also demonstrated that administration of TSA or a γ-secretase inhibitor RO4929097 in the mouse model of unilateral ureteral obstruction significantly ameliorated renal fibrosis through suppression of the JNK/Notch-2 signaling activation. Taken together, our findings provide further insights into the crosstalk among different signaling pathways in renal fibrosis, and elucidate the molecular action of TSA in attenuating fibrogenesis.

## Introduction

Renal fibrosis is the final pathological process common to all forms of chronic kidney disease^1^ and thereby represents an excellent treatment target. It is characterized by accumulation and activation of myofibroblasts, and extensive deposition of extracellular matrix in kidney parenchyma^2^. During development of renal fibrosis^2,3^, transforming growth factor-β1 (TGF-β1) is considered as the master mediator that induces myofibroblastic activation^4^ and abundant deposition of fibrotic matrix in renal tubulointerstitium. Although TGF-β1 signaling through the Smad-based (canonical) pathway^5–7^ is believed to play a critical role in the development of renal fibrosis, a growing body of evidence indicates that several non-Smad (non-canonical) pathways stimulated by TGF-β1 are also potentially involved in driving fibrosis in progressive kidney disease^8–10^. Among these TGF-β1-induced non-Smad signaling pathways, three major mitogen-activated protein kinases (MAPKs) pathways (including p38, ERK and JNK) have been suggested to contribute to inflammatory and fibrotic damages of various renal diseases^11–13^. Thus, detailed understanding the downstream networks of TGF-β1-mediated signaling during the progression of renal fibrosis would be helpful to develop new therapeutic strategies to prevent or delay kidney damage.

The Notch signaling pathway is an evolutionarily conserved pathway, which is known to play an essential role in renal development^14^. After completion of renal development, the Notch signaling pathway is largely suppressed^15^. In vertebrates, the Notch system consists of four highly conserved membrane receptors (Notch-1 to Notch-4) and five ligands (JAG-1, JAG-2, Delta-like-1, Delta-like-3, and Delta-like-4). Activation of Notch signaling pathway is initiated through the binding of ligands to Notch receptors. Upon ligand binding, the Notch receptor undergoes two consecutive proteolytic cleavages by ADAM metalloprotease and γ-secretase, ultimately leading to the release of the Notch intracellular domain (NICD). The resultant NICD then translocates into the nucleus, where it interacts with RBP-Jκ (also known as CSL or CBF-1) and Mastermind like-1 coactivator to cooperatively activate its downstream target genes, such as hairy enhancer of split (Hes) and Hes-related repressor (Hey) families^16^. Emerging evidence has shown that aberrant activation of the Notch signaling pathway could lead to epithelial-mesenchymal transition (EMT) and regulate interstitial fibrosis^17,18^. Murea *et al*., also reported that reactivation of Notch signaling in the tubulointerstitium correlates well with the severity of fibrosis and the decline of renal function^19^. In addition, genetic deletion of the Notch signaling in proximal tubular epithelial cells significantly reduced tubulointerstitial fibrosis in the mouse model of folic acid-induced nephropathy^17^. Currently, the functional interactions between Notch signaling pathway and the TGF-β1-mediated canonical or non-canonical pathways in renal fibrosis still remain elusive.

Trichostatin A (TSA), a histone deacetylase (HDACs) inhibitor that blocks both class I and II HDACs, has been demonstrated as a promising therapeutic agent in different forms of fibrotic diseases^20–24^. For example, TSA effectively inhibits EMT of hepatic stellate cells^20^ and abrogates TGF-β1-inducing fibrosis-related gene expression in skin fibroblasts^22^. Moreover, TSA has been shown to suppress EMT on retinal pigment epithelial cells by downregulating the Jagged/Notch signaling after TGF-β1 stimulation^23^. Importantly, Pang *et al*. have demonstrated that administration of TSA is able to inhibit myofibroblastic activation in cultured renal fibroblasts and attenuate renal fibrosis in mice with obstructive nephropathy^24^. Although TSA exerts an anti-fibrotic activity and attenuates the pathogenesis of renal injury in obstructive nephropathy, the underlying molecular basis of how TSA alleviates chronic kidney fibrotic injury is not fully understood.

In this study, we sought to investigate the relationship between Notch signaling pathway and TGF-β1-mediated canonical or non-canonical signaling pathways in the regulation of renal fibroblasts *in vitro* and *in vivo*. Additionally, we also tried to elucidate the molecular action of TSA in ameliorating renal fibrotic injury. On the basis of our findings *in vitro* and *in vivo*, we here propose that the JNK/Notch-2 signaling axis is critically involved in TGF-β1-mediated renal fibrosis, and TSA treatment efficiently suppresses the JNK-dependent Notch-2 signaling pathway.

## Results

### TGF-β1 upregulates profibrogenic factors and activates the Smad and the MAPKs signaling pathways in cultured rat renal fibroblasts

To establish an *in vitro* model of renal fibrosis, NRK-49F cells, a rat kidney interstitial fibroblast cell line, were first treated with increasing amounts of TGF-β1 (0, 1, 2 and 5 ng/ml) for 48 h. Western blot analysis showed that TGF-β1 at the dose of 5 ng/ml substantially increased levels of α-SMA and fibronectin, two hallmarks of activated fibroblasts, in treated cells (Supplementary Fig. S1a), and thus this concentration of TGF-β1 was suitable for subsequent experiments. Additionally, in time-course experiments, we found that the maximal induction of α-SMA was reached at 48 hr after treatment with TGF-β1 at 5 ng/ml (Supplementary Fig. S1b).

To further examine the activation of TGF-β1-mediated canonical or non-canonical signaling pathways in NRK-49F cells, short-term treatment of cells with TGF-β1 was performed. Western blot analysis revealed that TGF-β1 treatment rapidly induced phosphorylation of Smad2 and Smad3 in NRK-49F cells, and the levels of phospho-Smad2 and phospho-Smad3 reached a maximum at 30 and 60 min post-treatment, respectively (Supplementary Fig. S1c). Furthermore, TGF-β1 treatment also significantly increased the levels of phospho-p38, phospho-ERK and phospho-JNK in these treated cells, which reached a maximum at 90 min post-treatment (Supplementary Fig. S1d).

### Trichostatin A inhibits α-SMA up-regulation and JNK activation, but not the activation of Smad, p38 and ERK in TGF-β1-treated fibroblasts

**Figure 1:**
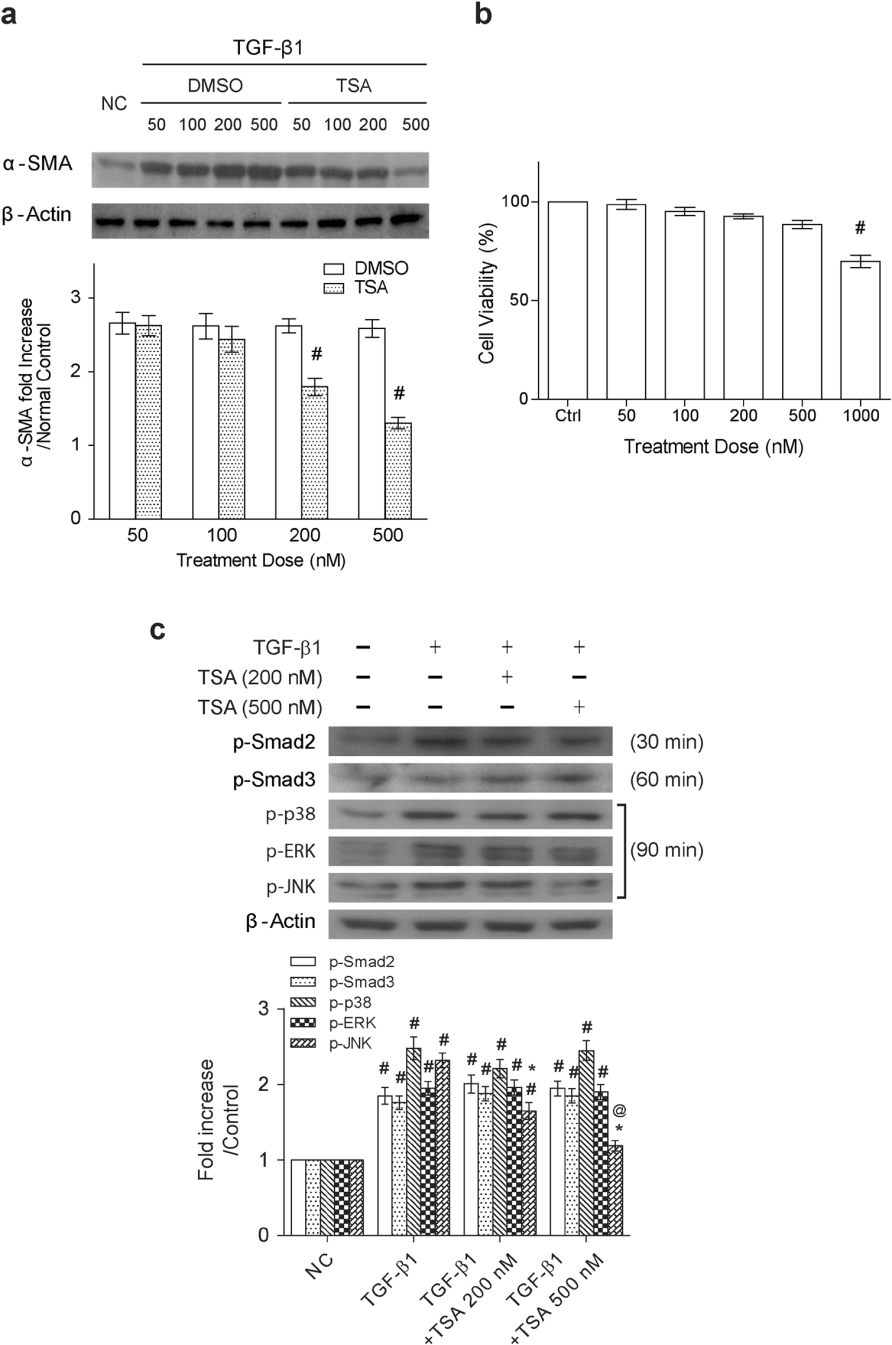
Trichostatin A inhibits α-SMA up-regulation and JNK activation, but not the activation of Smad, p38 and ERK in TGF-β1-treated fibroblasts. (**a**) Western blot analysis of α-SMA expression in NRK-49F cells that were treated with TGF-β1 (5 ng/ml) in combination with different concentrations of TSA or the solvent control (Dimethyl sulfoxide; DMSO) for 48 hr. Relative expression levels of α-SMA, normalized to β-actin, in the treated cells were quantified by densitometry (n = 6, bottom panel). Symbol # indicates significant difference vs. the control or vehicle group (P < 0.05). (**b**) Effects of TSA on cell viability of TGF-β1-treated NRK-49F cells. MTT assays were used to determine the cell viability and results were presented as means ± SEM calculated from at least three independent experiments. Symbol # indicates significant difference vs. the control group (P < 0.05). (**c**) Effects of TSA on the TGF-β1-mediated activation of Smad2, Smad3 and different MAP kinases. NRK-49F cells were treated with TGF-β1 in the presence or absence of TSA (200 or 500 nM), and the expression levels of phospho-Smad2 (at 30 min), phospho-Smad3 (at 60 min), phospho-p38 (at 90 min), phospho-ERK (at 90 min) and phospho-JNK (at 90 min) were examined in cell samples (n = 6). ^#^P < 0.05 vs. control group; *P < 0.05 vs. TGF-β1 group and ^@^P < 0.05 vs. the group treated with TGF-β1 plus TSA (200 nM).

In order to determine whether TSA inhibited TGF-β1-mediated fibrogenesis, NRK-49F cells were treated with the combination of TGF-β1 and TSA at different concentrations (50, 100, 200 and 500 nM). In agreement with a previous report^24^, we showed here that treatment of renal fibroblasts with TSA attenuated TGF-β1-mediated upregulation of α-SMA in a concentration-dependent manner (Fig. 1a). Particularly, TSA at 500 nM displayed potent suppressive activity on α-SMA upregulation, but had only a minor impact on cell proliferation in TGF-β1-treated NRK-49F cells (Fig. 1b). However, treatment of the solvent control dimethyl sulfoxide (DMSO) did not significantly affect α-SMA levels in TGF-β1-treated NRK-49F cells. Under such a condition, we also confirmed that a dose-dependent increase in acetylation of histones H3 and H4 was indeed observed in TSA-treated cells (Supplementary Fig. S2a).

Next, we sought to explore the molecular action of how TSA inhibits fibrogenesis caused by TGF-β1. Accordingly, NRK-49F cells that were exposed to TGF-β1 or the combination of TGF-β1 and TSA (200 or 500 nM) were harvested at different early time points (30, 60 and 90 min). The expression levels of phospho-Smad2, phospho-Smad3, and phospho-MAPKs were then analyzed in indicated cell groups harvested at 30, 60 and 90 min after treatment, respectively. Western blot analysis revealed that TSA predominantly abrogated TGF-β1-induced JNK phosphorylation, but not the phosphorylation of Smad2, Smad3, p38 and ERK (Fig. 1c).

### Notch-2 signaling is involved in TGF-β1-mediated myofibroblast activation

**Figure 2:**
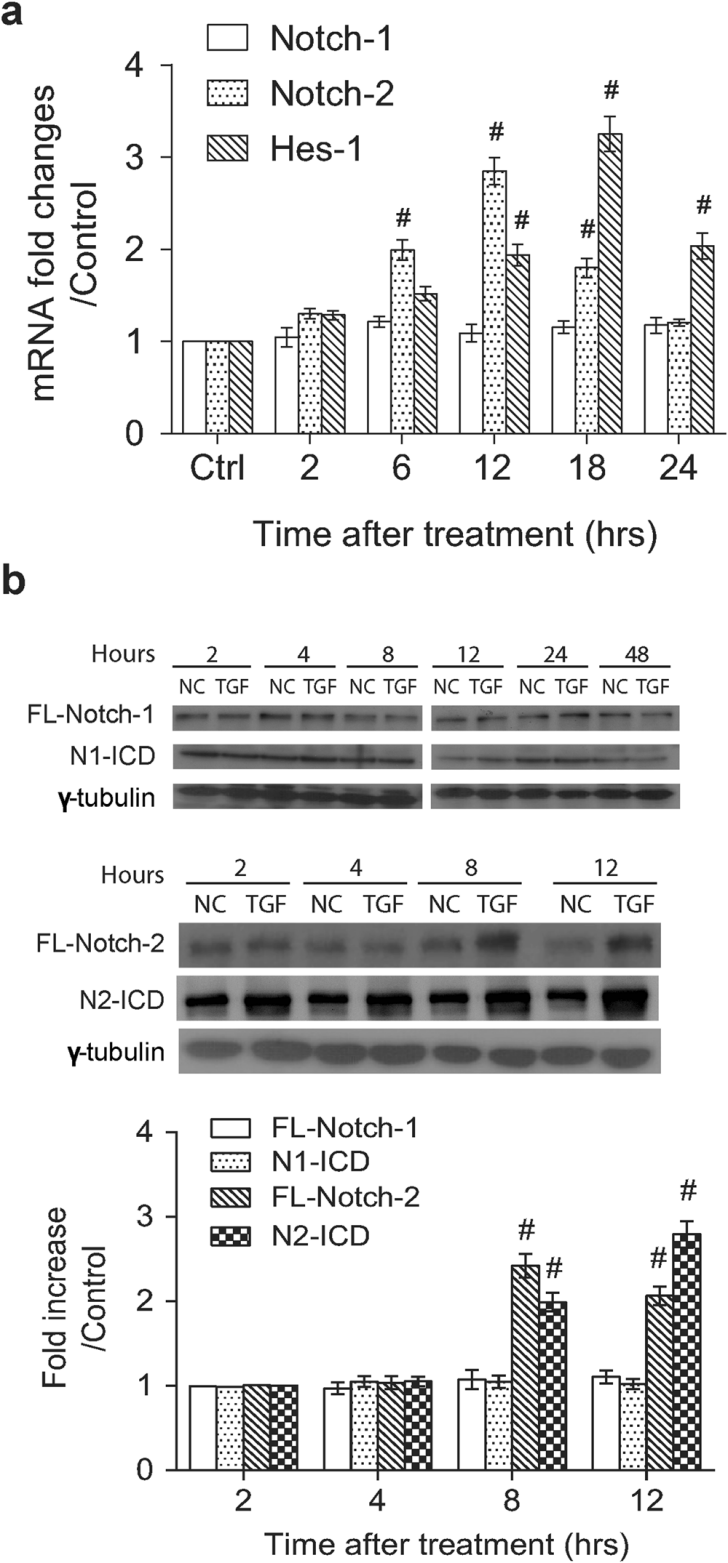
TGF-β1 specifically increases the expression levels of Notch-2, but not Notch-1, in rat renal fibroblasts. (**a**) NRK-49F cells were treated with TGF-β1 (5 ng/ml) for various periods of time. The mRNA expression levels of Notch-1, Notch-2 and Hes-1, normalized to that of β-actin, in these treated cells were quantified by qRT-PCR (n = 6). (**b**) Western blot analysis of full-length Notch-1, Notch-1 intracellular domain (N1-ICD), full-length Notch-2 and Notch-2 intracellular domain (N2-ICD) expressed in NRK-49F cells that were treated with TGF-β1 for different periods of time. Relative expression levels of the indicated proteins quantified by densitometry in the TGF-β1-treated cells were shown in the bottom panel. All experimental results from qRT-PCR or Western blotting are presented as means ± SEM (n = 6) and symbol # indicates significant difference vs. control group (P < 0.05).

To investigate whether TGF-β1 modulates Notch signaling in NRK-49F cells, we initially examined mRNA levels of Notch-1, Notch-2 and a downstream Notch target gene Hes-1. Quantitative RT-PCR analysis showed that TGF-β1 treatment substantially elevated the expression levels of Notch-2 and Hes-1 mRNAs, but not Notch-1 mRNA. In the qRT-PCR experiments, a significant increase in Notch-2 mRNA level was mainly observed within the time period between 6 and 18 hr after TGF-β1 treatment, whereas an evident increase in Hes-1 mRNA level occurred between 12 and 24 hr after treatment (Fig. 2a). Western blot analysis also demonstrated that TGF-β1 treatment increased the protein levels of the full-length Noch-2 and cleaved Notch-2 (N2-ICD), but not the expression of Notch-1 and cleaved Notch-1 (N1-ICD) (Fig. 2b).

**Figure 3:**
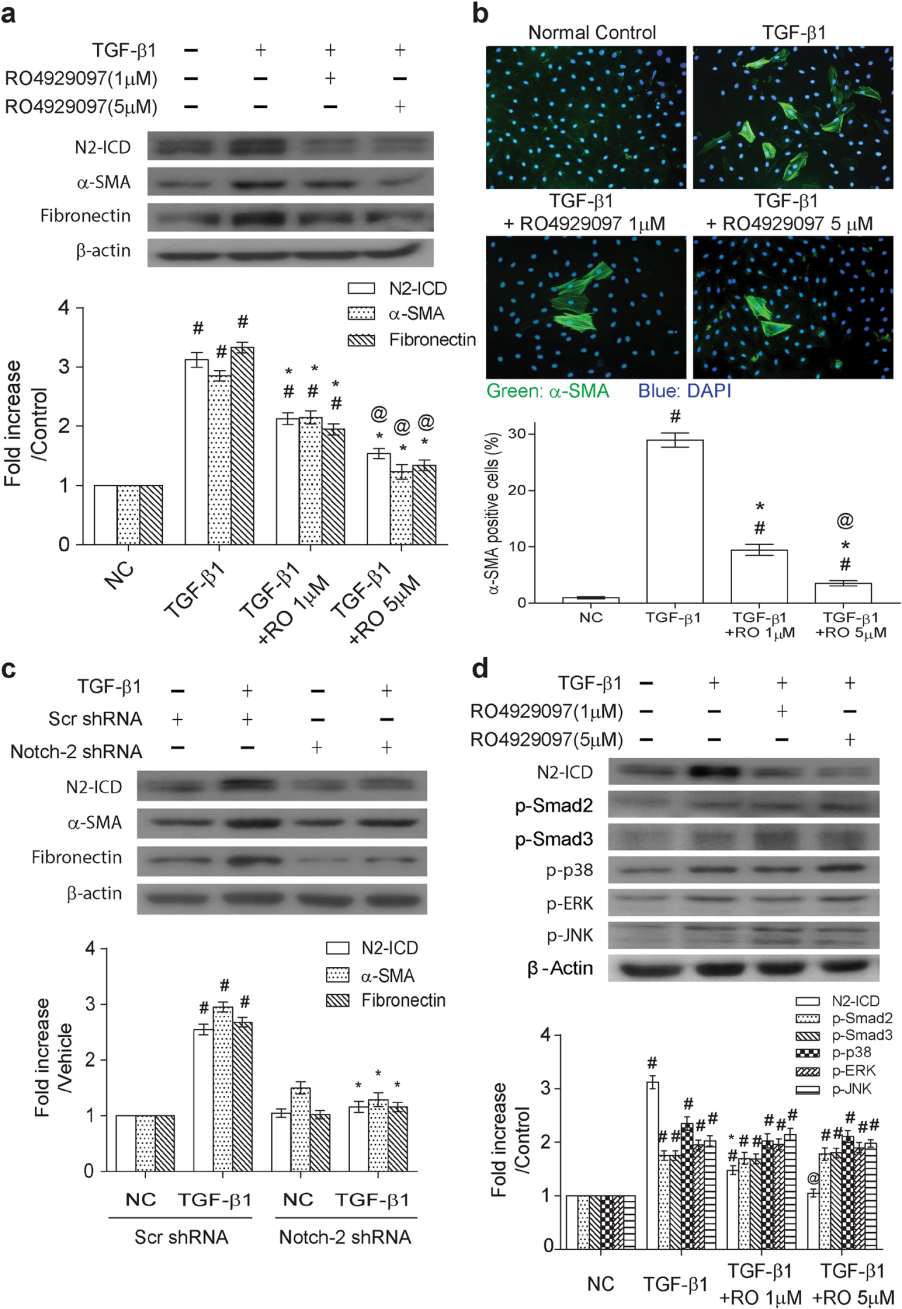
Notch-2 signaling is involved in TGF-β1-mediated myofibroblast activation. (**a**) NRK-49F cells were treated with TGF-β1 in the presence or absence of RO4929097 (1 or 5 μM). After 48 hr post-treatment, cells were subjected to Western blot analysis for N2-ICD, α-SMA and fibronectin (n = 6). ^#^P < 0.05 vs. control group; *P < 0.05 vs. TGF-β1 group and ^@^P < 0.05 vs. the group treated with TGF-β1 plus RO4929097 (1 μM). (**b**) Representative immunofluorescence images of the staining for α-SMA (green color) and 4’,6-diamidino-2-phenylindole (DAPI, blue color) in NRK-49F cells after exposure to TGF-β1 alone or the combination of TGF-β1 and RO4929097 (1 or 5 μM) for 48 hr. Proportions of α-SMA positive cells are shown in the bottom panel (n = 6). ^#^P < 0.05 vs. control group; *P < 0.05 vs. TGF-β1 group and ^@^P < 0.05 vs. the group treated with TGF-β1 plus RO4929097 (1 μM). (**c**) NRK-49F cells were transiently transfected with the plasmid expressing Notch-2 shRNA or scrambled shRNA, and the transfected cells were then treated with TGF-β1 for 48 hr. Protein levels of α-SMA and fibronectin were examined by Western blot analysis (n = 6). ^#^P < 0.05 vs. control group; *P < 0.05 vs. TGF-β1 group. (**d**) Effects of RO4929097, a γ-secretase inhibitor, on activation of Smad2, Smad3, p38, ERK and JNK in the TGF-β1-treated cells. ^#^P < 0.05 vs. control group; *P < 0.05 vs. TGF-β1 group and ^@^P < 0.05 vs. the group treated with TGF-β1 plus RO4929097 (1 μM). Results from densitometric analysis are presented as means ± SEM from at least three experiments.

To explore whether the increased Notch-2 signaling critically contributed to myofibroblastic activation, a γ-secretase/Notch signaling pathway inhibitor RO4929097 was used in the study. Addition of the γ-secretase inhibitor RO4929097 in TGF-β1-treated NRK-49F cells critically reduced not only the N2-ICD level, but also levels of α-SMA and fibronectin expression (Fig. 3a). Consistently, results from immunofluorescence assays also revealed that RO4929097 substantially diminished the proportions of α-SMA-positive cells in TGF-β1-stimulated NRK-49F cells (Fig. 3b). Moreover, knockdown of N2-ICD using the plasmid expressing Notch-2 shRNA efficiently blocked upregulation of α-SMA and fibronectin expression caused by TGF-β1 in NRK-49F cells (Fig. 3c). To further investigate the potential association between Notch-2 signaling and Smad or MAPKs signaling, effects of RO4929097 on the levels of phospho-Smad2, phospho-Smad3, phospho-p38, phospho-ERK and phospho-JNK were evaluated in NRF-49F cells after TGF-β1 simulation. Although RO4929097 treatment efficiently reduced N2-ICD levels in NRK-49F cells after TGF-β1 stimulation, RO4929097 treatment did not influence phosphorylation of Smad2, Smad3, p38, ERK and JNK in TGF-β1-treated cells (Fig. 3d). These results demonstrated that Notch-2 signaling is critically involved in TGF-β1-induced myofibroblast activation, and probably functions downstream of the Smad or the MAPK signaling cascades.

### TSA inhibits TGF-β1-mediated Notch-2 activation through inactivation of JNK

**Figure 4:**
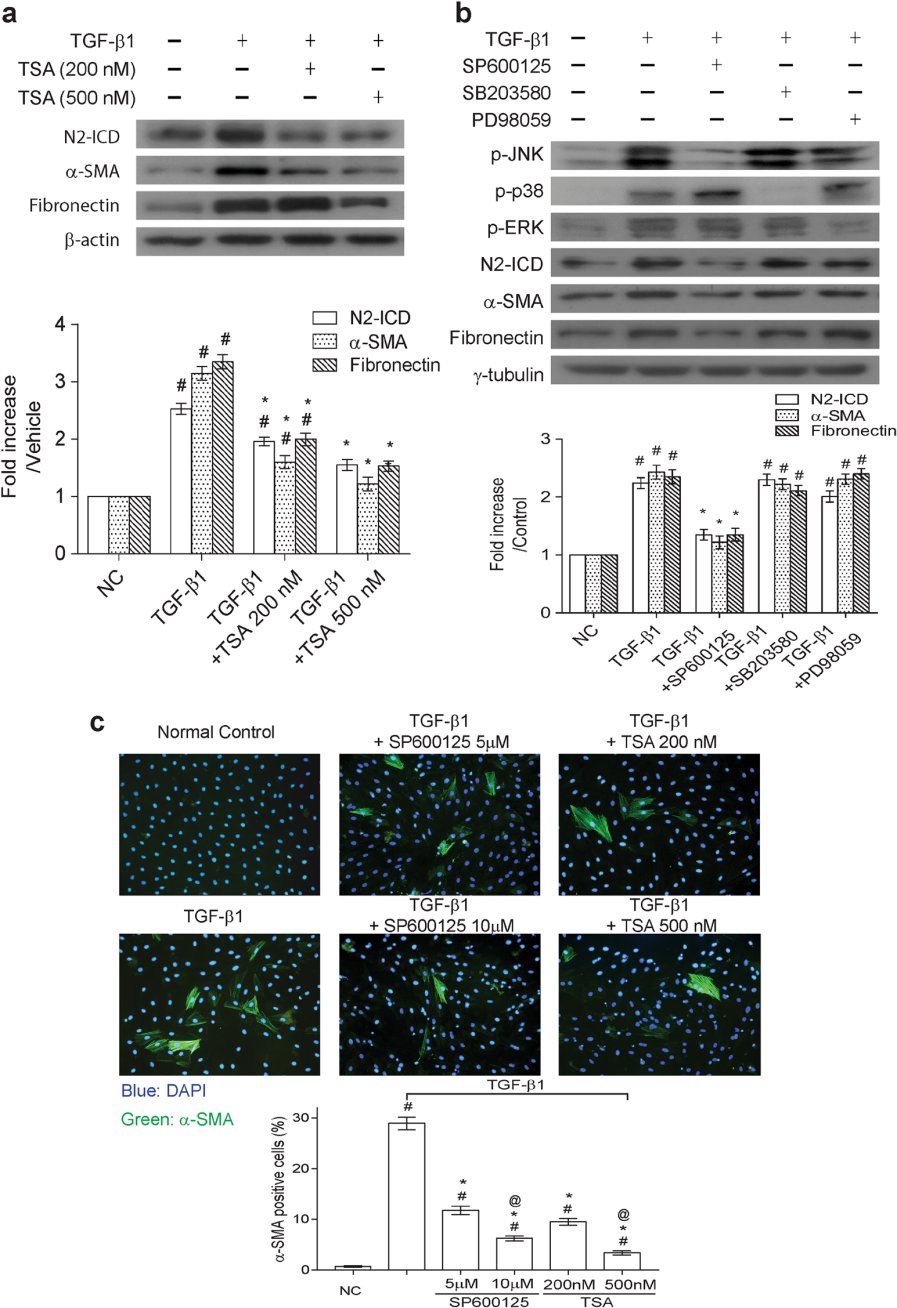
Treatment of NRK-49F cells with trichostatin A or SP600125 (a JNK inhibitor) alleviates the TGF-β1-mediated myofibroblastic induction through modulating the Notch-2 signaling pathway. (**a**) Effect of TSA on the TGF-β1-mediated activation of Notch-2, α-SMA and fibronection. Western blot analysis was performed using cell lysates of NRK-49F cells that were treated with 5 ng/ml TGF-β1 in combination with TSA at 200 or 500 nM for 48 hr. (**b**) Effects of MAPK (JNK, p38 or ERK) inhibitors on TGF-β1-mediated myofibroblastic activation. NRK-49F cells were concurrently treated with TGF-β1 and 10 μM SP600125, 10 μM SB203580 or 20 μM PD98059 for 48 hr. Protein expression of phospho-JNK, phospho-p38, phospho-ERK, N2-ICD, α-SMA and fibronectin was determined by Western blot analysis. Results obtained from densitometric analysis are presented as means ± SEM from at least three experiments. ^#^P < 0.05 vs. control group; *P < 0.05 vs. TGF-β1 group. (**c**) Representative immunofluorescence images of α-SMA (green color) staining in NRK-49F cells. The nuclei were counterstained with DAPI (blue color). Treatment of TGF-β1-treated cells with SP600125 or TSA dose-dependently reduced the formation of α-SMA positive cells. Proportions of α-SMA positive cells are shown in the bottom panel (n = 6). ^#^P < 0.05 vs. control group; *P < 0.05 vs. TGF-β1 group and ^@^P < 0.05 vs. the group treated with TGF-β1 plus SP600125 (5 μM) or TGF-β1 plus TSA (200 nM), respectively.

As shown above, both TSA and RO4929097 had the same action to reduce the upregulation of profibrogenic factors caused by TGF-β1 in NRK49-F cells (Figs 1a and 3). We then assessed whether TSA affected Notch-2 activation in TGF-β1-treated NRK49-F cells. Results from Western blot analysis displayed that TSA indeed blocked both activation of Notch-2 and upregulation of profibrogenic factors in TGF-β1-treated cells (Fig. 4a). Due to the observations that TSA specifically inhibited JNK activation (Fig. 1c), we further clarified the causal relationship between Notch-2 signaling and the activation of different MAPKs in TGF-β1-treated renal fibroblasts. Different pharmacological blockers for p38 (SB203580, 10 μM), ERK (PD98059, 20 μM) and JNK (SP600125, 10 μM) were utilized to block individual signaling cascade in the experiments (Fig. 4b). We found that treatment with the JNK inhibitor SP600125 markedly attenuated the level of cleaved Notch-2 in the TGF-β1-stressed NRK-49F cells. However, treatment with the p38 inhibitor SB203580 or the ERK inhibitor PD98059 did not show discernible effects on the expression of cleaved Notch-2 in the TGF-β1-stimulated NRK-49F cells. Furthermore, only SP600125 treatment effectively blocked upregulation of α-SMA and fibronectin caused by TGF-β1 (Fig. 4b). Consistently, immunofluorescence analysis also showed that SP600125 treatment reduced the proportions of α-SMA-expressing myofibroblasts in TGF-β1-stimulated cells (Fig. 4c). Taken together, these results strongly suggest that TSA targets JNK-dependent Notch-2 activation to attenuate TGF-β1-mediated fibrogenesis in renal fibroblasts.

### TSA or RO4929097 attenuates tubulointerstitial fibrosis in mice with unilateral ureteral obstruction (UUO)

**Figure 5:**
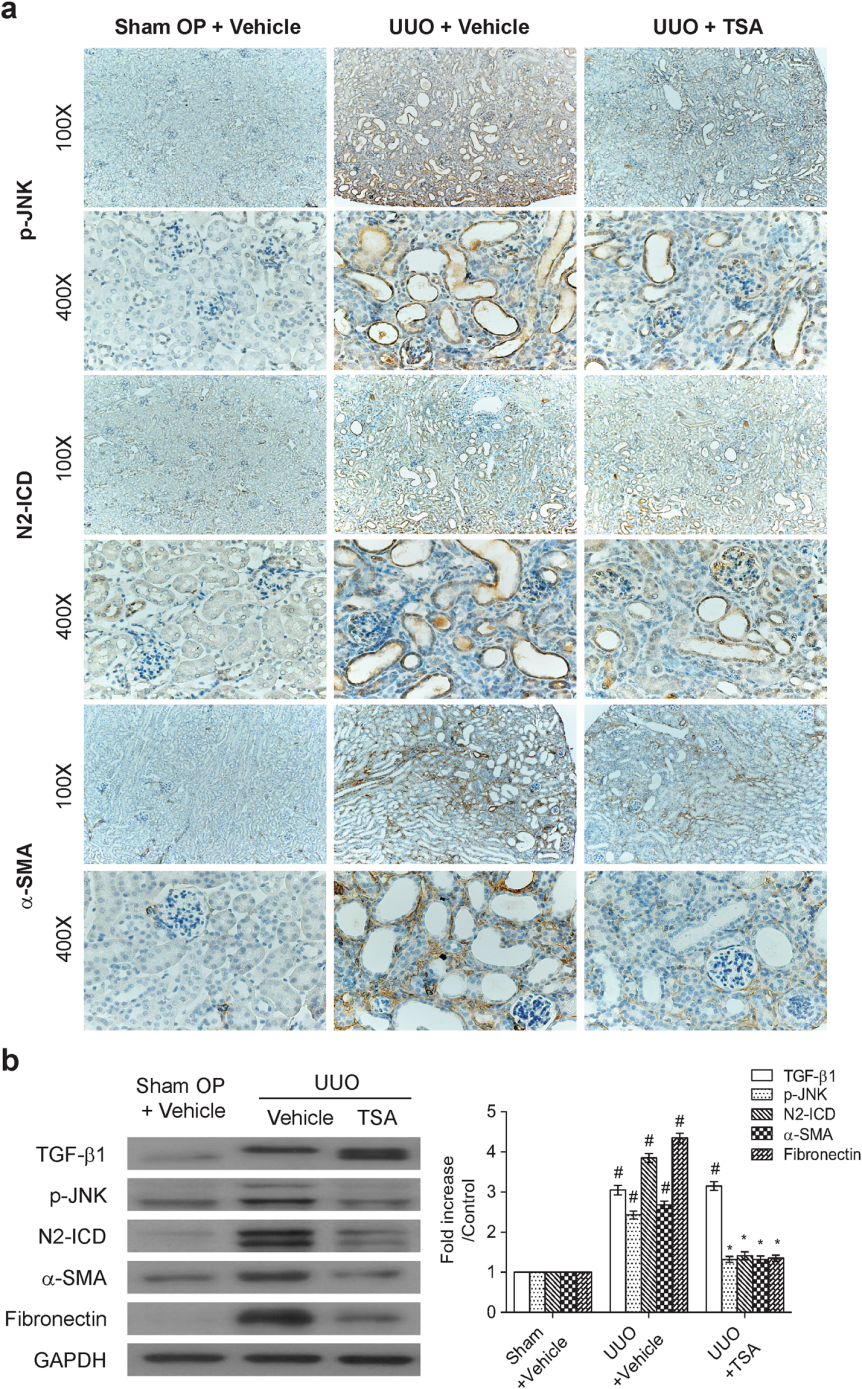
Trichostatin A prevents the activation of JNK and N2-ICD and the expression of α-SMA and fibronectin in mice with unilateral ureteral obstruction (UUO). (**a**) Representative photographs of phospho-JNK, N2-ICD and α-SMA immunohistochemical staining in the renal cortex of normal kidneys and UUO kidneys with or without intraperitoneal injection of TSA (500 μg/kg/day from day 1 to day 6). Magnifications: × 100 and × 400. (**b**) TSA treatment abolished the increased phospho-JNK, N2-ICD, α-SMA and fibronectin expression detected in the UUO renal tissue protein lysates as determined by Western blot analysis. Data are presented as means ± SEM calculated from at least three independent experiments. ^#^P < 0.05 vs. sham operated group; *P < 0.05 vs. UUO mice treated with vehicles group.

**Figure 6:**
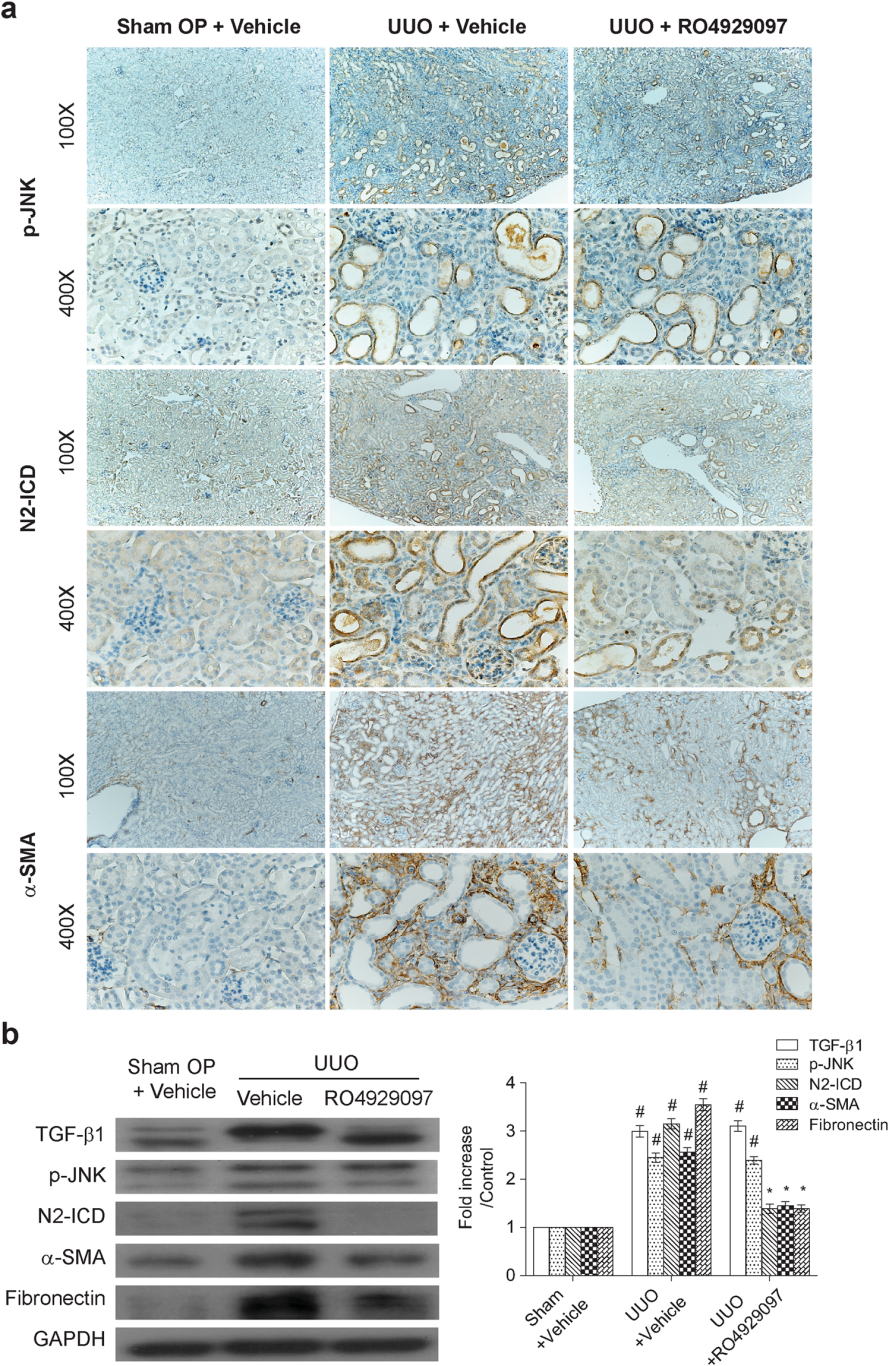
γ-secretase inhibitor, RO4929097, downregulated Notch-2 and profibrogenic gene expression in UUO kidneys. (**a**) Representative photographs of the immunohistochemical staining for phospho-JNK, N2-ICD and α-SMA in the renal cortex of the sham operated mice and UUO mice with or without oral gavage of RO4929097 (5 mg/kg/day from day 1 to day 6). Magnifications: × 100 and × 400. (**b**) Treatment of RO4929097 reduced Notch-2, α-SMA and fibronectin activation in UUO kidneys. Protein lysates from renal tissues were subjected to Western blot analysis of TGF-β1, phospho-JNK, N2-ICD, α-SMA and fibronectin. Data from densitometric analysis are presented as means ± SEM calculated from at least three experiments. ^#^P < 0.05 vs. sham operated group; *P < 0.05 vs. UUO mice treated with vehicles group.

**Figure 7:**
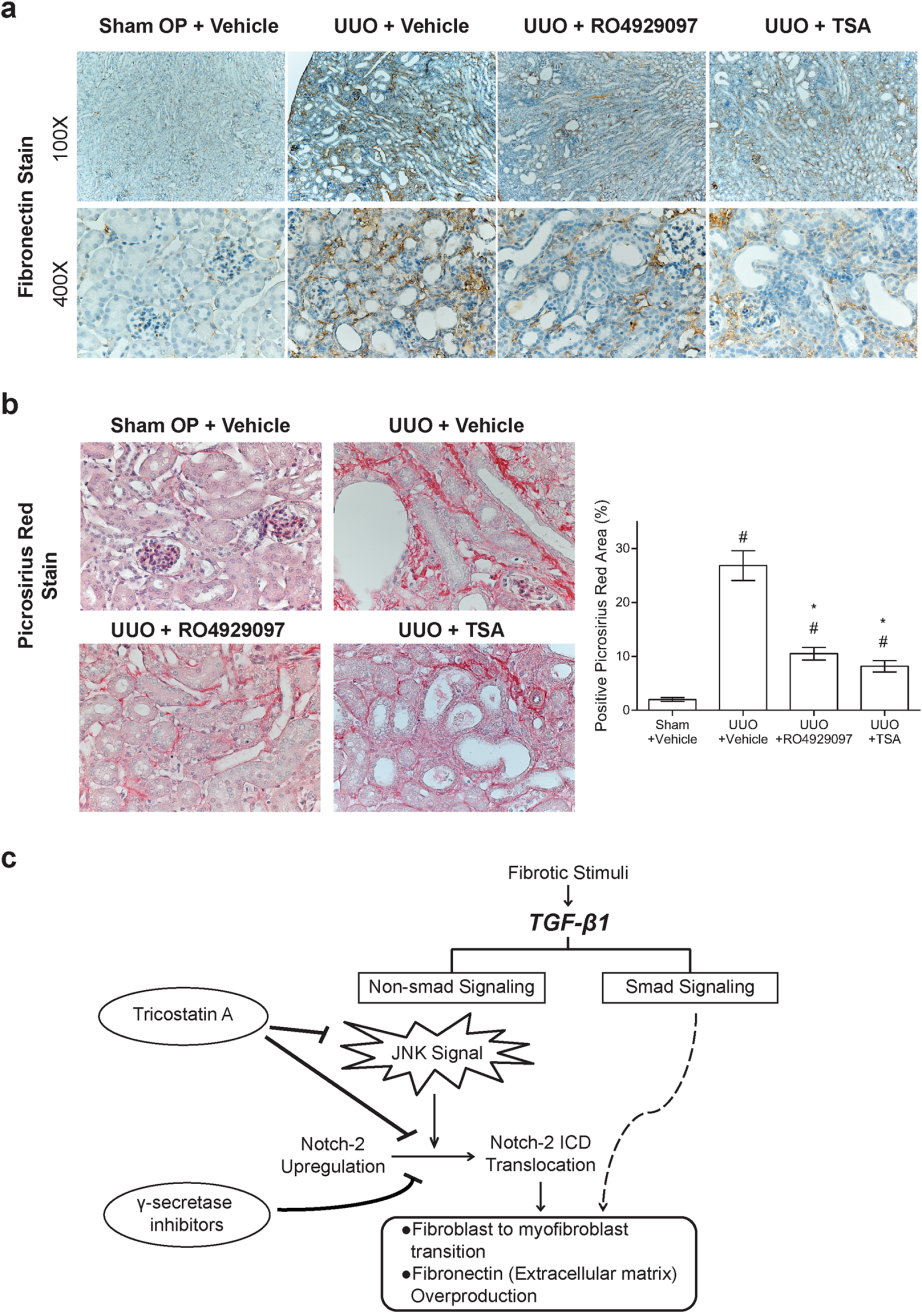
Trichostatin A and γ-secretase inhibitor ameliorates tubulointerstitial fibrosis in UUO mice. (**a**) Immunohistochemical staining for fibronectin in the renal cortex samples of the sham operated mice and UUO mice treated with the drug vehicle, RO4929097 and TSA. Magnifications: × 100 and × 400. (**b**) Picrosirius red fibrosis staining of renal cortex under light microscopy (magnification × 400). There was increased amount and branching of collagen fibers in UUO mice treated with vehicles. Treatment of TSA or RO4929097 significantly diminished the picrosirius-positive staining area as shown in the bar chart derived from densitometric quantification (n = 6). ^#^P < 0.05 vs. sham operated group; *P < 0.05 vs. UUO mice treated with vehicles group. (**c**) Proposed model of the JNK-dependent Notch-2 signaling in renal fibrotic damage.

To validate our conclusions from our *in vitro* model system, we then investigated whether TSA or RO4929097 had an anti-renal fibrotic activity in mice with unilateral ureteral obstruction (UUO). In the experiments, mice were divided into the sham-operated group, UUO group, and the UUO group that received daily treatment with TSA (500 μg/kg) or RO4929097 (5 mg/kg). Kidney samples were harvested at day 7 after sham or UUO operation for further analysis. Immunohistochemical staining analysis revealed that kidney tissue samples from UUO group displayed stronger staining and more positive cells for phospho-JNK, cleaved Notch-2, α-SMA and fibronectin in tubulointerstitial areas as compared to those from the sham-operated group (Figs 5a, 6a and 7a). Consistent with the immunohistochemical analysis, Western blot analysis also showed that the protein levels of TGF-β1, phospho-JNK, cleaved Notch-2, α-SMA and fibronectin in UUO group were significantly higher than those in sham-operated group (Figs 5b and 6b). However, administration of TSA or RO4929097 markedly suppressed the levels of cleaved Notch-2, α-SMA and fibronectin as detected by immunostaining and immunoblotting analyses (Figs 5–7). Regarding the action of TSA, we concurrently confirmed that TSA could significantly increase the amount of acetylated histones H3 and H4 in the mice model (Supplementary Fig. S2b). As noted, RO4929097 blocked only Notch-2 activation, but not JNK activation, in UUO-treated mice (Fig. 6). Additionally, using picrosirius red staining, we also found that treatment with TSA or RO4929097 substantially attenuated collage deposition in obstructed kidneys by 69 and 59%, respectively (Fig. 7b). Noteworthily, neither TSA nor RO4929097 reduced UUO-induced TGF-β1 expression (Figs 5b and 6b). Additionally, similar to our *in vitro* studies, we did not detect significant changes in the expression level of cleaved Notch-1 in kidney samples among the sham-operated group, UUO group, and the UUO group receiving TSA or RO4929097 treatment (Supplementary Fig. S3).

## Discussion

TGF-β1 is well recognized as the key mediator that critically drives fibroblast activation and matrix deposition during development of renal fibrosis. In addition to the canonical (Smad-dependent) signaling pathway, TGF-β1 also induces a variety of non-canonical (Smad-independent) signaling pathways to promote renal fibrosis. In this report, we identified that the JNK/Notch-2 signaling axis is the major arm of the non-canonical TGF-β1 signaling pathways involved in fibrogenesis in cultured renal fibroblasts and in UUO-induced mice. Additionally, we also showed that TSA, a HDAC inhibitor, effectively attenuates renal fibrogenesis mainly through inhibiting the JNK/Notch-2 signaling activation both *in vitro* and *in vivo* (Fig. 7c). Better understanding of various downstream pathways of TGF-β1-mediated signaling and the potential crosstalk of these pathways may help to provide alternative therapeutic strategies to block renal fibrosis.

By using cultured renal fibroblasts (NRK-49F cells), we here showed that TGF-β1 treatment efficiently induced myofibroblastic activation (α-SMA upregulation) and matrix deposition (fibronectin upregulation) (Supplementary Fig. S1a and b). At the early time points after TGF-β1 treatment, both canonical (Smad2/3-based) and non-canonical (including p38, JNK and ERK) signaling pathways were also activated in NRK-49F cells (Supplementary Fig. S1c and d). Following the activation of Smad2/3, p38, JNK and ERK, we detected a concomitant increase in the full-length and cleaved Notch-2 as well as Hes-1, but not the full-length and cleaved Notch-1 (Fig. 2). Specially, the mRNA expression of Notch-2 induced by TGF-β1 reached its maximum at 12 hr post-treatment (Fig. 2a), whereas the protein expression of full-length Notch-2 was detected with the maximal level at 8 hr and slightly declined at 12 hr (Fig. 2b). Interestingly, cleaved Notch-2 continued to rise during the period between 2 and 12 hr after TGF-β1 treatment (Fig. 2b). It is therefore possible that TGF-β1 concurrently stimulated γ-secretase activity and accelerated the cleavage of full-length Notch-2 into N2-ICD, thereby resulting in the decline of full-length Notch-2 protein earlier than we expected. Although previous studied have shown that aberrantly activated Notch signaling in chronic kidney disease is strongly related to the development of renal fibrosis, the impact and activation of individual Notch receptors in renal fibrosis still remains controversial and actually varies in different experimental models of kidney disease. For example, the increased level of cleaved Notch-1 was predominantly found in tubules of folic acid-induced nephropathy^17^, spontaneously hypertensive rats^25^ and diabetic nephropathy^19^, whereas upregulation of cleaved Notch-2 was reported in those of focal segmental glomerulosclerosis^19^ and ischemic reperfusion injury^26^. Notch-2, rather than Notch-1, plays an important role in generating tubulointerstitial fibrosis in our myofibroblast and UUO models. It is therefore possible that activation of Notch receptors, especially for Notch-1 and Notch-2, in the development and progression of renal fibrosis could be in a context-dependent, cell-type-specific manner.

To further determine the importance of Notch-2 activation in TGF-β1-mediated fibrogenesis, pharmacological inhibition or genetic knockdown of Notch-2 was applied in our *in vitro* experiments. Block of Notch-2 activation by the γ-secretase inhibitor RO4929097 or by Notch-2 shRNA significantly downregulated the expression of α-SMA and fibronectin in TGF-β1-treated fibroblasts (Fig. 3a–c), supporting that Notch-2 activation is indeed required for promoting fibrogenesis in renal fibroblasts. Due to the observation that inhibition of Notch signaling by RO492907 did not influence activation of Smad2/3, p38, JNK and ERK in TGF-β1-treated NRK-49F cells, the Notch-2 activation could be a downstream event of these signaling pathways. Interestingly, after block of each branch of MAPKs signaling using inhibitors, we found that the JNK inhibitor SP600125 sufficiently blocked Notch-2 activation, which was accompanied by downregulation of α-SMA and fibronectin in TGF-β1-treated fibroblasts (Fig. 4a and c). To our knowledge, this is the first report showing that JNK acts as an upstream regulator of Notch-2 signaling in TGF-β1-stressed renal fibroblasts. In our mouse model of UUO, we also consistently found that inactivation of the JNK/Notch-2 signaling axis by TSA (discussed more fully below) or by RO4929097 markedly attenuated renal fibrotic injury in obstructed kidneys (Figs 5–7). All our experiments highlight the importance of the alternative JNK/Notch-2 signaling axis in renal fibrogenesis.

TSA, a potent and reversible HDACs inhibitor, has been reported to have anti-fibrotic activities in murine models of UUO^24,27^, nephrotoxic serum nephritis^28^ and adriamycin nephropathy^29^. Some reports have suggested that TSA might target TGF-β1/Smad pathway to attenuate renal fibrotic injury^20,23,30,31^. However, in our *in vitro* model using renal fibroblatic cells NRK-49F, we did not find that TSA influenced the phosphorylation status of Smad2 and Smad3 in TGF-β1-stressed cells (Fig. 1), despite potent inhibition of both α-SMA and fibronectin upregulation by TSA (Figs 1 and 4). For the non-canonical TGF-β1 signaling pathways, the critical roles of MAPK signaling cascades in TGF-β1-induced profibrogenic responses, including EMT and collagen synthesis, have already been established in cultured renal cells^32,33^. Additionally, individual branch of MAPK signaling pathways that potentially contribute to renal fibrosis was also suggested in diabetic patients, streptozotocin-induced type 1 diabetic mice^11^, or other experimental animal models^34–36^. In our present study, we found that treatment of TSA only suppressed JNK activation, but not the activation of p38 and ERK in TGF-β1-stimulated NRK-49F cells (Fig. 1c). The suppression of JNK activation by TSA also concurrently led to inactivation of Notch-2 and downregulation of fibrogenic factors in TGF-β1-stimulated renal fibroblasts and in UUO mice (Figs 1, 5 and 7). These results strongly support that specific inhibition of the JNK/Notch-2 signaling activation by TSA relevantly ameliorates renal fibrogenesis.

In conclusion, data from our experiments demonstrate that upregulated Notch-2, at least for renal fibroblasts, plays a pivotal role in the pathological process of renal fibrosis. Pharmacological inhibition of JNK suppresses TGF-β1-induced activation of Notch-2 signaling. Furthermore, we elucidate that TSA treatment reduces myofibroblast activation and attenuates tubulointerstitial fibrosis through modulating the non-Smad pathway (Fig. 7c). Thus, these findings implicate that manipulation of JNK-dependent Notch-2 pathway may provide a potential therapeutic approach for halting progression of renal fibrosis. Especially, RO4929097 is a more selective and orally available inhibitor of γ-secretase. Currently, RO4929097 is well tolerated in clinical phase I/II anti-cancer trials^37,38^, which may make it possible in the treatment of chronic kidney disease.

## Materials and Methods

### Cell culture and treatments

Normal rat kidney fibroblast cells (NRK-49F, from American Type Culture Center, Manassas, VA) were cultured in Dulbecco’s Modified Eagle Medium supplemented with 2 mM L-glutamine, 1% non-essential amino acids and 5% fetal bovine serum (Gibco, Carlsbad, CA) in a 5% CO_2_, 37 °C humidified incubator. To examine the pro- or anti-fibrotic effects of TGF-β1 and TSA, subconfluent cultures of NRK-49F cells were first maintained quiescent in basal medium with 0.5% fetal bovine serum for 24 hrs, and then stimulated with 5 ng/ml TGF-β1 (PeproTech, Rocky Hill, NJ) in the presence or absence of TSA (Sigma-Aldrich, St Louis, MO). To investigate the roles of MAPKs and Notch signaling pathways in TGF-β1-mediated fibrogenesis, subconfluent NRK-49F cells were treated with the following inhibitors: 10 μM SB203580 **(**p38 inhibitor; Gibco, Frederick, MD), 20 μM PD98059 (ERK inhibitor; Gibco, Frederick, MD), 10 μM SP600125 (JNK inhibitor; Gibco, Camarillo, CA) and RO4929097 (γ-secretase inhibitor; Selleckchem, Houston, TX).

### Quantitative reverse transcription polymerase chain reaction (qRT-PCR)

Total RNAs were extracted from cells by using QIAzol lysis reagent (Qiagen, Valencia, CA) according to the manufacturer’s instructions. One microgram of total RNA was reverse-transcribed into cDNA with the ReverAid^TM^ M-MuLV reverse transcriptase (Fermentas, Glen Burnie, MD). Each qPCR mixture (25 μL) contained cDNA templates (equivalent to 20 ng of total RNA), 2.5 μM of each forward and reverse primers, and 2X iQ^TM^ SYBR Green Supermix (Bio-Rad, Hercules, CA), and reactions were performed on an iCycler iQ Real-time PCR Detection System (Bio-Rad Laboratories, Hercules, CA) with an initial melting at 95 °C for 5 minutes followed by 35 cycles of denaturation at 94 °C for 15 seconds, annealing at 52 °C for 20 seconds, and extension at 72 °C for 30 seconds. The PCR primer sets used in this study were following: 5′-GCTACAACTGCGTGTGTGTC and 5′-GTTGGTGTCGCAGTTGGAGC for Notch-1; 5′-CACCTTGAAGCTGCAGACAT and 5′-GGTAGACCAAGTCTGTGATG for Notch-2; 5′-GGTGGCTGCTACCCCAGCCA and 5′-GGTAGGTCATGGCGTTGATC for Hes-1; 5′-CGCCAACCGCGAGAAGAT and 5′-CGTCACCGGAGTCCATCA for β-actin. All quantitative RT-PCR experiments were performed in duplicate from at least three independent treatments. The relative gene expression was calculated as described previously^39^.

### Cell proliferation/ viability assay

To determine the drug toxicity and optimal treatment dose, NRK-49F cells cultured under different doses of TSA were evaluated with tetrazolium-based colorimetric MTT assay (Roche, Mannheim, Germany). Briefly, NRK-49F cells (1 × 10^4^ cells/well, 96-well) were cultured in the absence or presence of TSA (50, 100, 200, 500 and 1000 nM) for 48 hrs, and then 3-(4,5-dimethythiazol-2-yl)-2,5-diphenyl tetrazolium bromide (10 μl/well) were added to the cells and incubated for another 4 hrs. Formazan crystals in each well were resolved by 100 μl 10% sodium dodecyl sulfate (SDS)-0.01 M HCl. Colorimetric changes were measured with a microplate reader (Molecular Devices Corp., Sunnyvale, CA) at wavelength of 450–500 nm.

### Protein extraction and Western blotting analyses

Total cellular proteins were extracted from renal tissues and fibroblast cells as described previously^40^. Equal aliquots of lysates were fractionated on 8–12% acrylamide gel and the blots were probed with primary antibodies against acetylated histone H3 (Ac-H3), histone H4 (Ac-H4), full-length Notch-1, cleaved Notch-1, full-length Notch-2, cleaved Notch-2, phosphorylated p38, p38, phosphorylated ERK, ERK, phosphorylated JNK, JNK, phosphorylated Smad2, phosphorylated Smad3, Smad2/3 (Cell Signaling Technology, Beverly, MA), TGF-β1 (Santa Cruz Biotechnology, Dallas, TX), α-SMA and fibronectin (Abcam, Cambridge, UK), followed by horseradish peroxidase-conjugated IgG (Santa Cruz Biotechnology, Dallas, TX) as the secondary antibody, and visualized by chemiluminescence. These membranes were stripped and then reprobed with β-actin, γ-tubulin (Cell Signaling Technology, Beverly, MA) or GAPDH (Santa Cruz Biotechnology, Dallas, TX) with the same procedures to show the equal loading. The relative intensities of immunoblot signals were measured by densitometry using Image-Pro Plus image analysis software (SNAP-Pro *c*.*f*. Digital kit; Media Cybernetics Inc., Silver Spring, MD) and were expressed as fold changes relative to vehicle.

### Knockdown of Notch2 using short hairpin RNAs (shRNAs)

Plasmids expressing Notch-2 shRNA (5′-TGAGGACTCTTCTGCCAACAT-3′) and a scrambled shRNA (5′-GGAATCTCATTCGATGCATAC-3′) were purchased from Qiagen (Duesseldorf, Germany). Transient transfection experiments were performed using the Lipofectamine 2000 Transfection Reagent (Invitrogen, Carlsbad, CA) according to the manufacturer’s instructions. After transfection for 48 hr, green fluorescent protein (GFP) expressed from the transfected plasmids was used as a marker to determine efficiency of transfection. Notch-2 knockdown was verified by Western blotting analysis (Fig. 3c).

### Immunofluorescence

Cells cultured on sterile coverslips in six-well plates were fixed with 4% paraformaldehyde for 15 min at room temperature, permeabilized with 0.1% Triton X-100 in phosphate buffered saline (PBS) for 15 min, and blocked with 4% bovine serum albumin in PBS for 30 min. The cells were then incubated with primary antibody against α-SMA (mouse IgG, 1:100 dilution) (Abcam, Cambridge, UK) overnight at 4 °C, followed by incubation with a secondary antibody (FITC-conjugated rabbit anti-mouse IgG, 1:1000 dilution) (Santa Cruz Biotechnology, Dallas, TX) at 37 °C for one hour in the dark. The cell nuclei were stained with 4’,6-diamidino-2-phenylindole (DAPI, 1:500 dilution; Santa Cruz Biotechnology, Dallas, TX). The slides were then visualized using a fluorescence microscope (Carl Zeiss, Jena, Germany).

### Unilateral ureteral obstruction (UUO) animal model

Seven-week-old male C57BL/6 mice (BioLasco Biotechnology Co., Taiwan) weighing 20 g were used and fed with a standard laboratory diet and water ad libitum. After induction of general anesthesia, the UUO surgery was carried out by creating an incision in the left flank and ligating both the proximal and distal left ureter with a 4–0 silk suture. Mice in the sham operation group underwent the same surgical procedure, but the ureter was not ligated. At day 7 after surgery, the mice were euthanized by cervical dislocation and kidney tissues and blood samples were harvested for further analyses.

To study the effects of TSA on the progression of renal fibrosis in UUO mice, eighteen mice were randomly divided into three groups, including the sham group (n = 6), the UUO group (n = 6), and the TSA-treated UUO group (n = 6). Mice from the TSA-treated UUO group received daily intraperitoneal injection of TSA (500 μg/kg/day) from Day 1 to Day 6. To further investigate the pro-fibrotic role of Notch signaling, UUO mice were received with RO4929097 at a dose of 5 mg/kg/day in 0.1 ml/oral gavage (n = 6), or were fed with vehicle (n = 6) consecutively from Day 1 to Day 6. In the experiments, the sham-operated mice served as normal controls (n = 6). All animal experiments were approved by the Institutional Animal Care and Use Committee of Chang Gung Memorial Hospital, and were performed in accordance with the Animal Protection Law by the Council of Agriculture, Executive Yuan (R.O.C.) and the guideline of National Research Council (U.S.A.) for the care and use of laboratory animals.

### Immunohistochemistry

Kidney tissues were fixed in 4% PBS buffered formaldehyde, embedded in paraffin, and then sliced longitudinally into 4-μm thick sections. Antibodies against phosphorylated JNK (R & D Systems, Minneapolis, MN), cleaved Notch-1, cleaved Notch-2, α-SMA and fibronectin (Abcam, Cambridge, UK) were used for immunohistochemistry. Immunoreactivity in sections was detected using a horseradish peroxidase-3′-,3′-diaminobenzidine kit (SuperPicture™ Kit, Invitrogen, Carlsbad, CA).

### Evaluation of tubulo-interstitial fibrosis by picrosirius red staining

The Picrosirius Red Stain Kit (Polysciences, Warrington, PA) was used for the histological evaluation of tubulointerstitial fibrosis. Sections were examined by polarized light microscopy. Photographs of ten random cortical fields (×400) from each animal sample were taken using a Cool CCD camera (SNAP-Pro *c*.*f*. Digital kit; Media Cybernetics, Sliver Spring, MD, USA). The positive-stained area of picrosirisus red was measured utilizing an ImagePro Plus image analysis software.

### Statistical Analysis

All values present in the study were means ± standard errors. For *in vitro* studies, data were measured and calculated from at least three independent experiments. One-way analysis of variance followed by Bonferroni post hoc test was performed to compare the differences among the groups. P < 0.05 was considered to be statistically significant.

### Data Availability

The datasets generated during and/or analyzed during the current study are available from the corresponding author on reasonable request.

## Electronic supplementary material

Supplementary Information
