# Supplementary material for: Trichostatin A ameliorates renal tubulointerstitial fibrosis through modulation of the JNK-dependent Notch-2 signaling pathway
Source: Sci Rep. 2017 Nov 3;7:14495. doi: 10.1038/s41598-017-15162-6 (PMC5670251; doi:10.1038/s41598-017-15162-6)
Supplement: Supplementary file 1 — Supplementary Information [file 41598_2017_15162_MOESM1_ESM.pdf]

Supplementary Information

Trichostatin A ameliorates renal tubulointerstitial fibrosis through modulation of the JNK-dependent Notch-2 signaling pathway

Chun-Wu Tung[1,3,4], Yung-Chien Hsu[1,4], Chang-Jhih Cai[1], Ya-Hsueh Shih[1,4], Ching-Jen Wang[6.7], Pey-Jium Chang[1,3*] & Chun-Liang Lin[1,2,4,5,6,*]

[1]Departments of Nephrology, Chang Gung Memorial Hospital, Chiayi, Taiwan; [2]Colleage of Medicine, and [3]Graduate Institute of Clinical Medical Sciences, Chang Gung University, Taiwan; [4]Kidney and Diabetic Complications Research Team (KDCRT), Chang Gung Memorial Hospital, Chiayi, Taiwan; [5]Kidney Research Center, Chang Gung Memorial Hospital, Taipei, Taiwan; [6]Center for Shockwave Medicine and Tissue Engineering, Kaohsiung Chang Gung Memorial Hospital and Chang Gung University College of Medicine, Kaohsiung, Taiwan; [7]Department of Orthopedic Surgery, Chang Gung Memorial Hospital, Kaohsiung, Taiwan

*Correspondence and requests for materials should be addressed to C.-L.L. (email: linchunliang@cgmh.org.tw) and P.-J.C. (email: peyjiumc@mail.cgu.edu.tw)

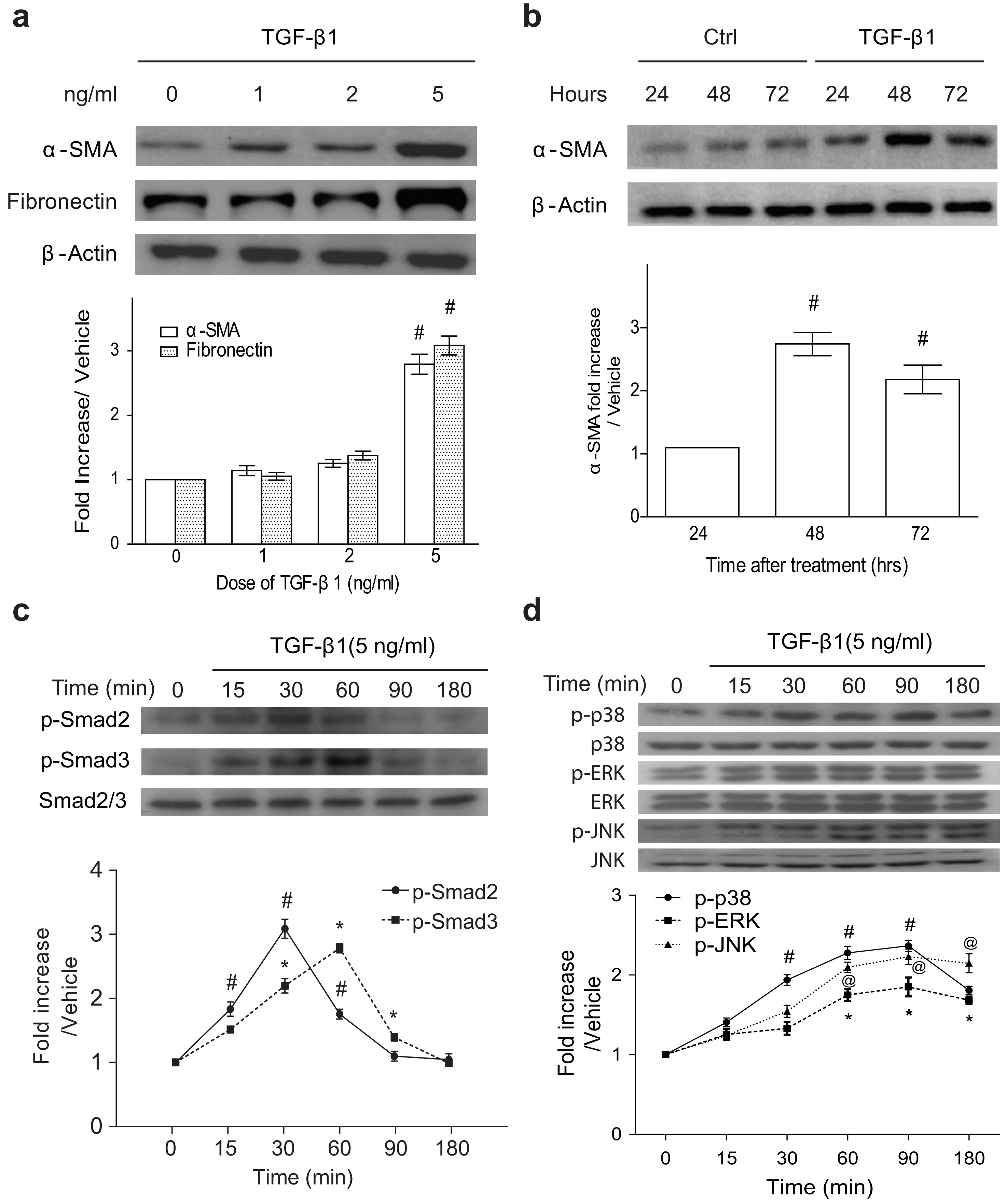

Figure S1. Transforming growth-β1 (TGF-β1) upregulates profibrogenic factors and activates the Smad and the MAPKs signaling pathways in cultured rat renal fibroblasts. (**a**) Rat kidney interstitial fibroblasts (NRK-49F cells) were cultured with increasing amounts of TGF-β1 (0, 1, 2 and 5 ng/ml) for 48 hr. The protein expression of α-SMA and fibronectin was determined by immunoblotting (top panel) followed by densitometric quantification (n=6, bottom panel). (**b**) Western blot analysis of α-SMA expression in NRK-49F cells treated with TGF-β1 (5 ng/ml) or vehicle for 24, 48 and 72 h. (**c** and **d**) Activation of Smad2, Smad3, p38, ERK and JNK by TGF-β1 in NRK-49F cells. Relative levels of the phosphorylated Smad2, Smad3, p38, ERK and JNK at the indicated time points were measured and shown in graphs. Symbols #, * and @ indicate a significant difference ($P < 0.05$) for different target proteins in comparison with the control group. Data are means ± SEM of at least three independent experiments.

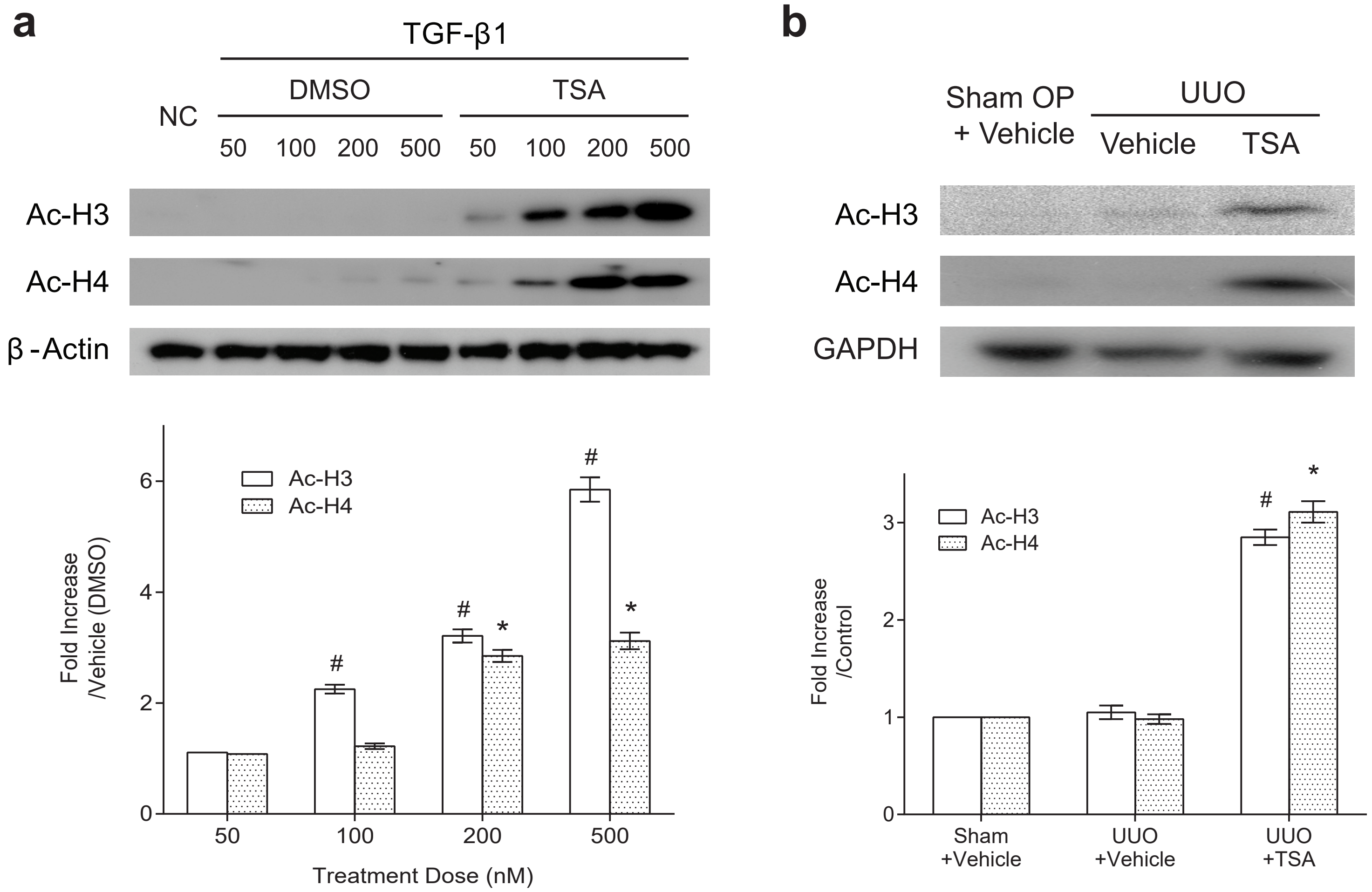

Figure S2. Trichostatin A treatment induces levels of acetylated histone H3 (Ac-H3) and H4 (Ac-H4) *in vitro* and *in vivo*. (**a**) NRK-49F cells treated with TGF-β1 (5 ng/ml) in combination with different concentrations of TSA or DMSO for 24 hr were subjected to Western blot analysis. Relative protein expression levels of Ac-H3 and Ac-H4, normalized to that of β-actin, were quantified by densitometric analysis from at least three independent experiments. Symbol # and * indicates significant difference vs. the control group ($P < 0.05$). **(b)** TSA treatment significantly increased expression of Ac-H3 and Ac-H4 in UUO mice as determined by immunoblotting analysis. Data from densitometic analysis (bottom panel) are presented as means ± SEM from at least three independent experiments. Symbol # and * indicates $P < 0.05$ vs. sham operated group. .

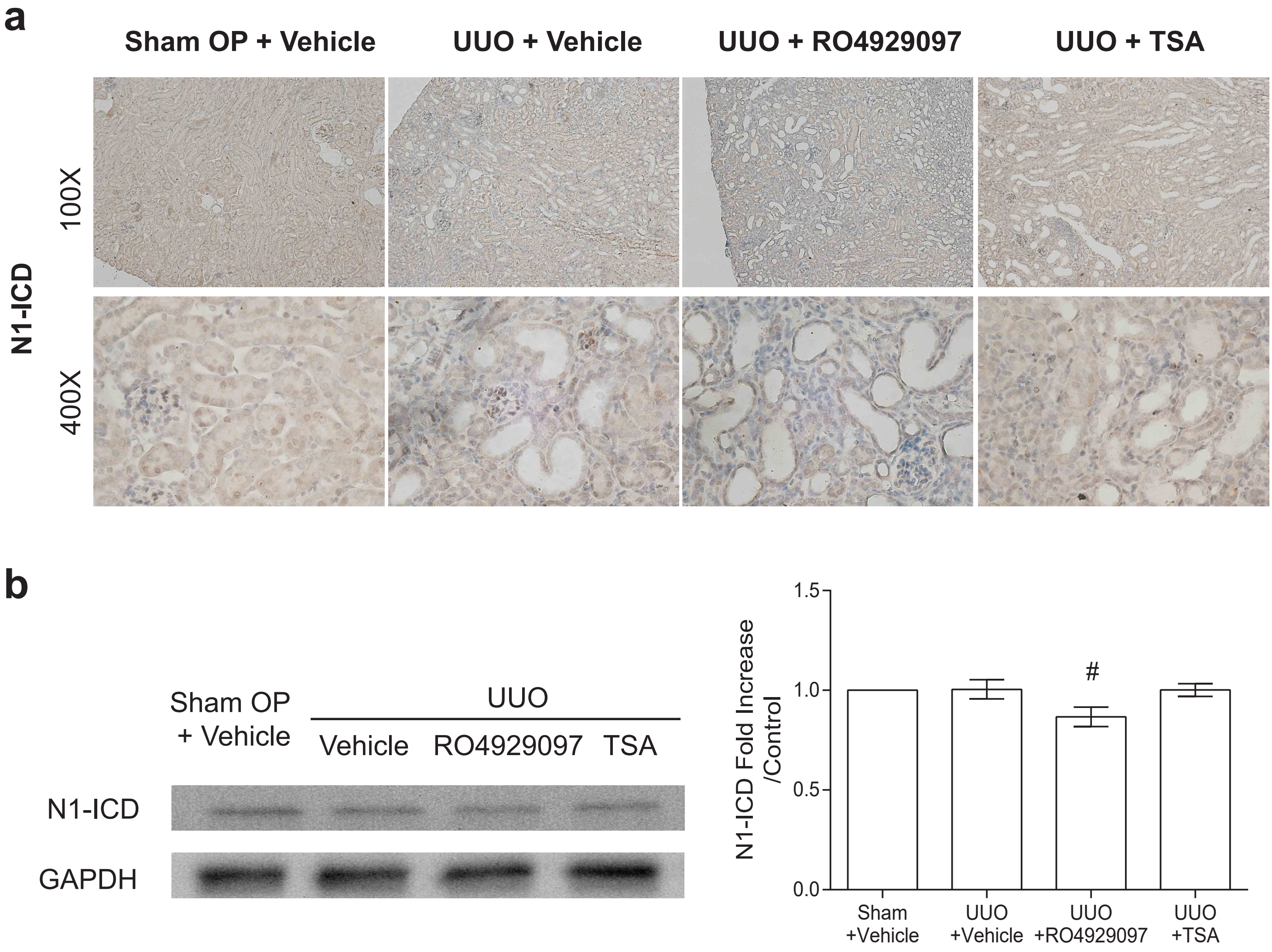

Figure S3. (**a**) Representative photographs of the immunohistochemical staining for Notch-1 intracellular domain (N1-ICD) in the renal cortex of sham operated mice, UUO mice treated with drug vehicles, UUO mice treated with RO4929097 and UUO mice treated with TSA. Magnifications: ×100 and ×400. (**b**) Protein lysates from renal tissues were subjected to Western blot analysis of N1-ICD and GAPDH. Results obtained from densitometric analysis are presented as means ± SEM from at least three independent experiments. #P< 0.05 vs. sham operated group

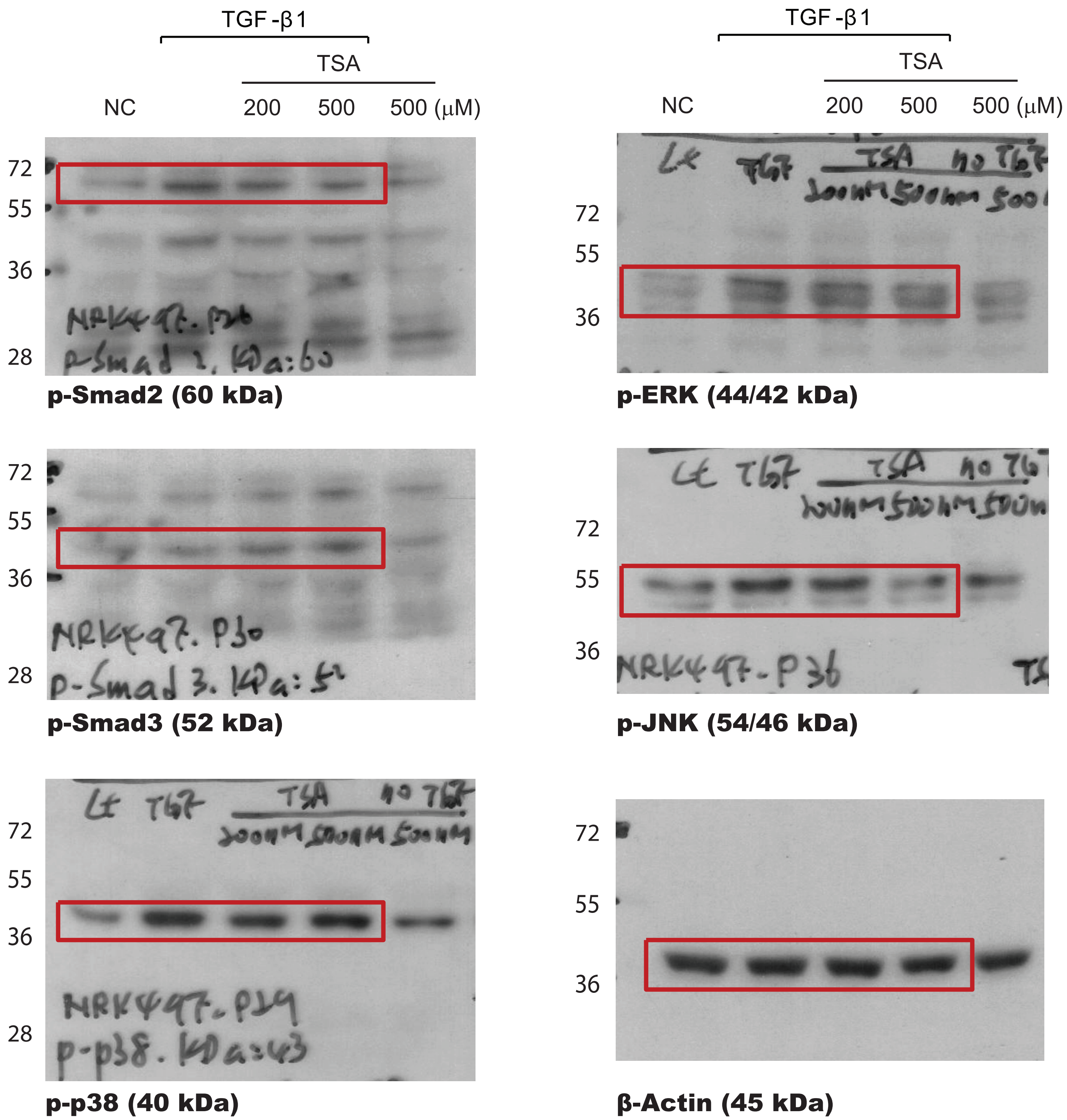

Figure S4, Full Length Immunoblotting images for Fig. 1c.

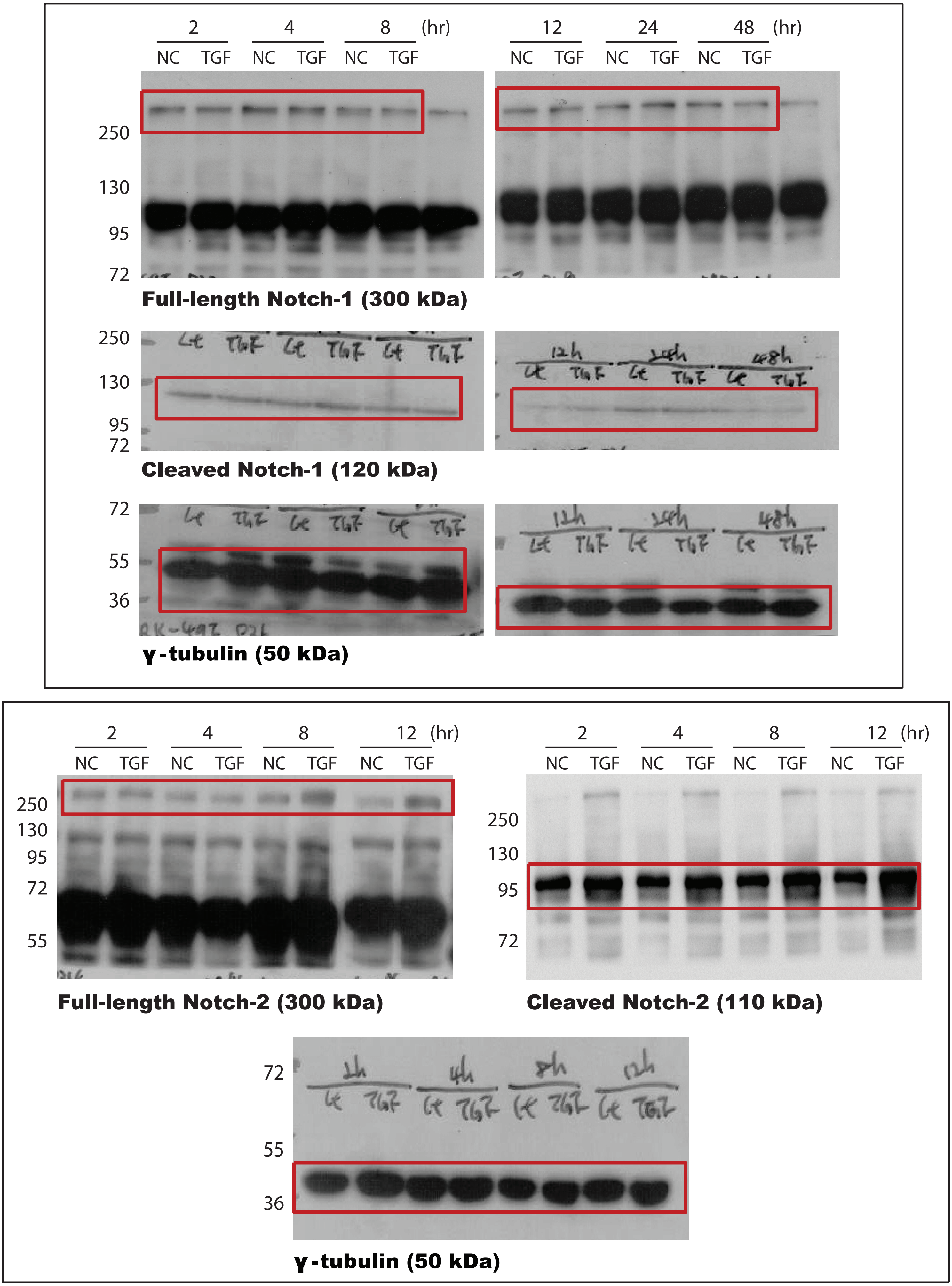

Figure S5, Full Length Gel images for Fig. 2b.

Fig. 3a

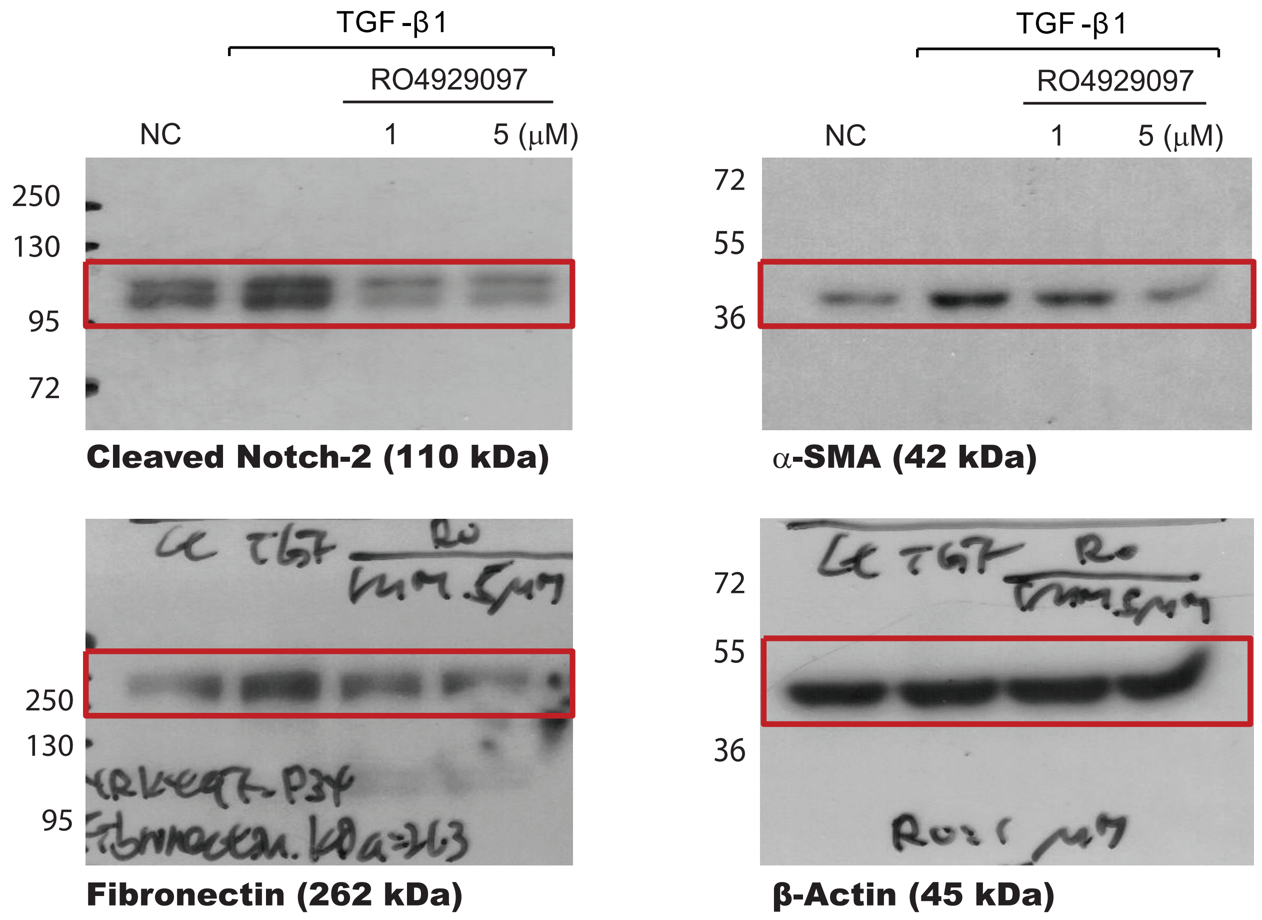

Fig. 3c

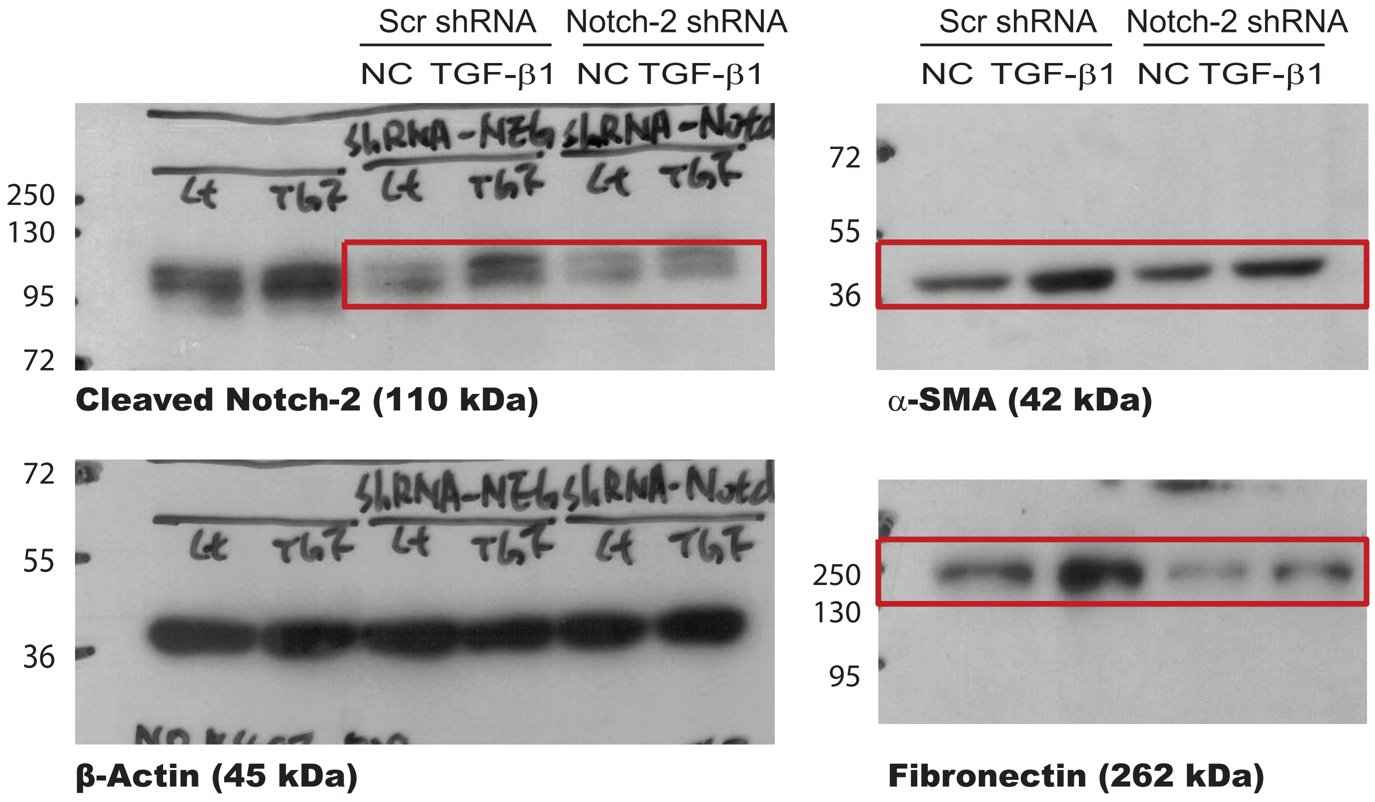

Figure S6, Full Length Western-blot images for Fig. 3a and 3c.

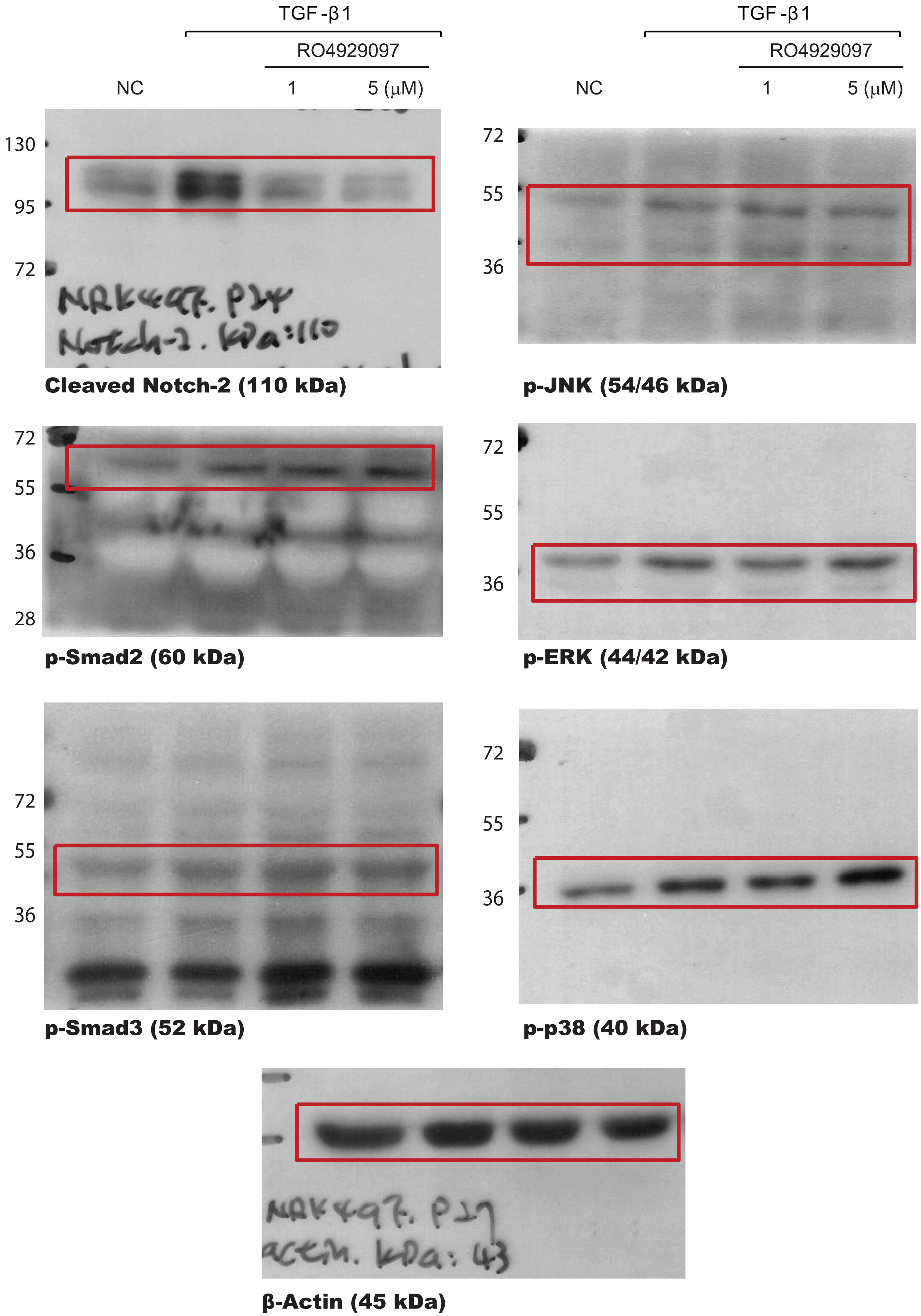

Figure S7, Full Length Gel images for Fig. 3d.

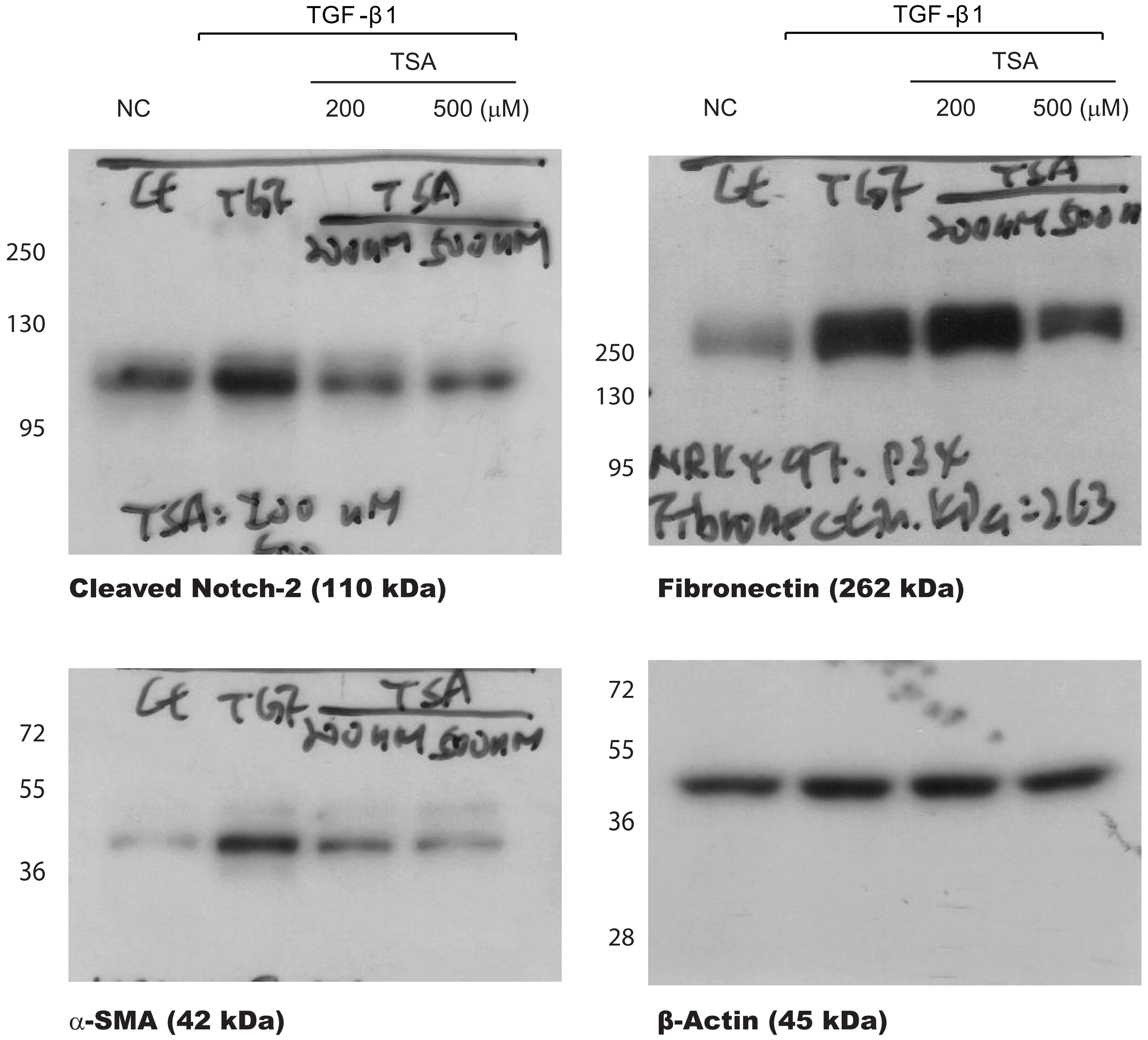

Figure S8, Full Length Gel images for Fig. 4a.

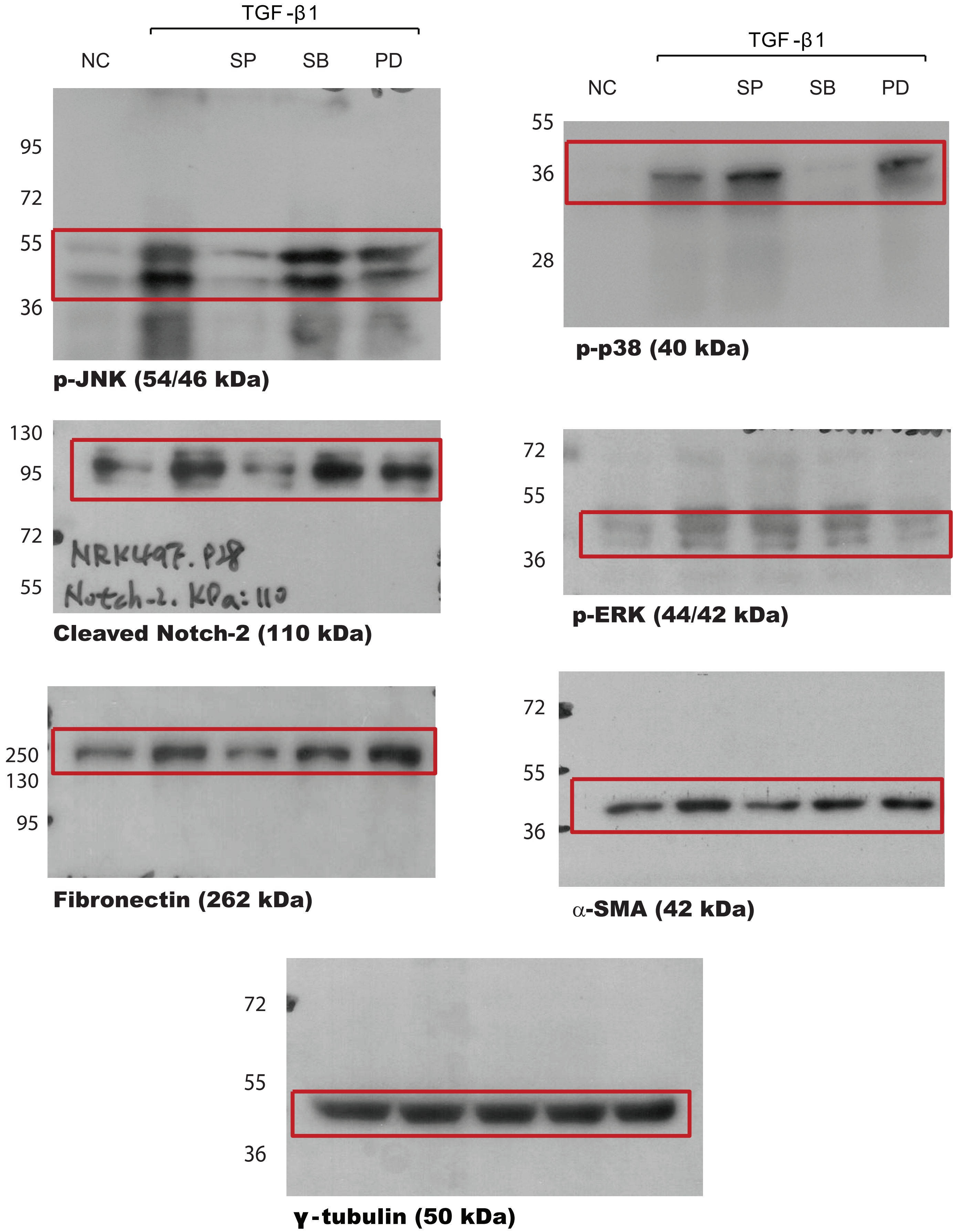

Figure S9, Full Length Gel images for Fig. 4b. (SP: SP600125; SB: SB203580; PD: PD98059)

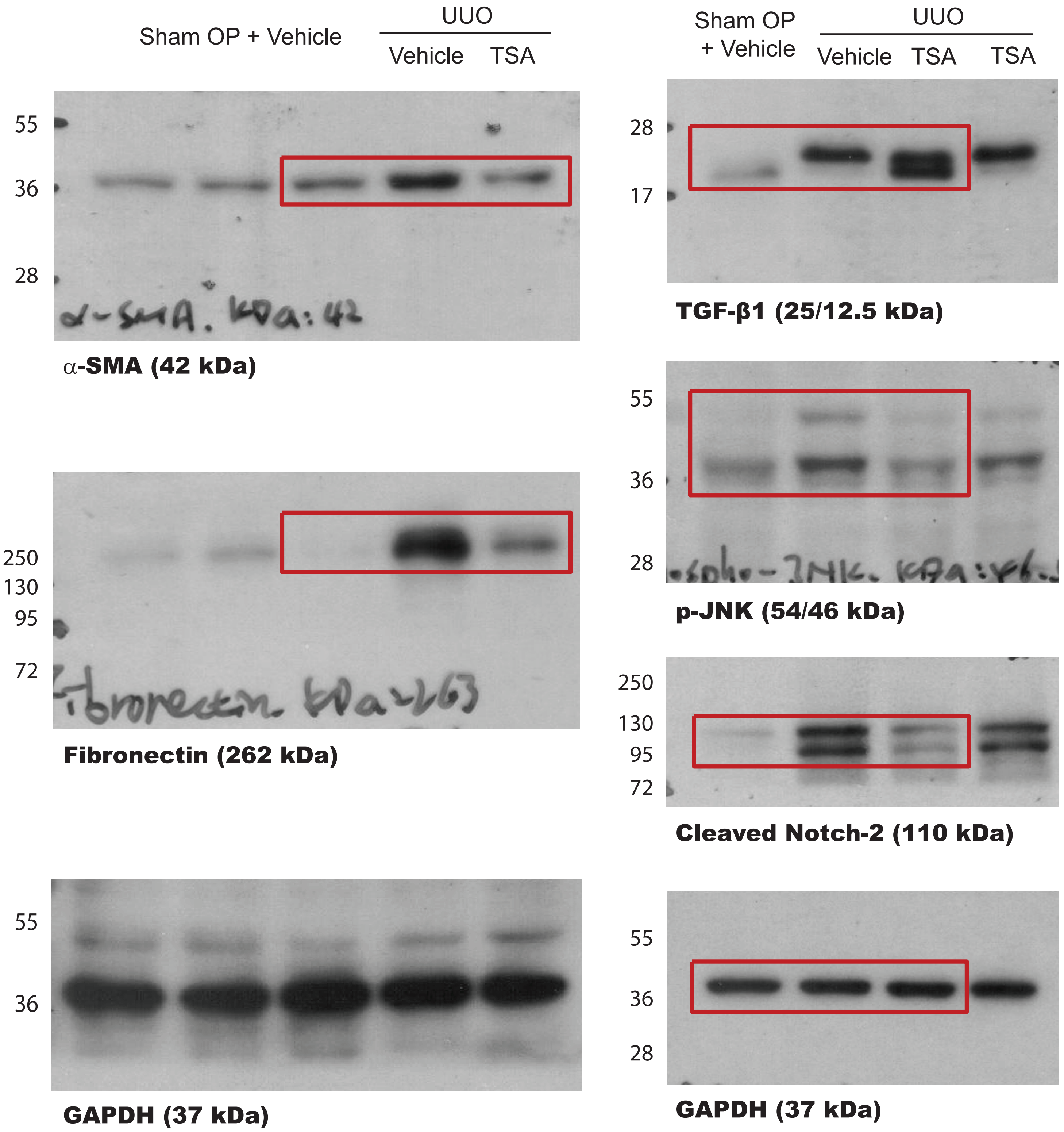

Figure S10, Full Length Western-blot images for Fig. 5b. (TSA: Trichostatin A)

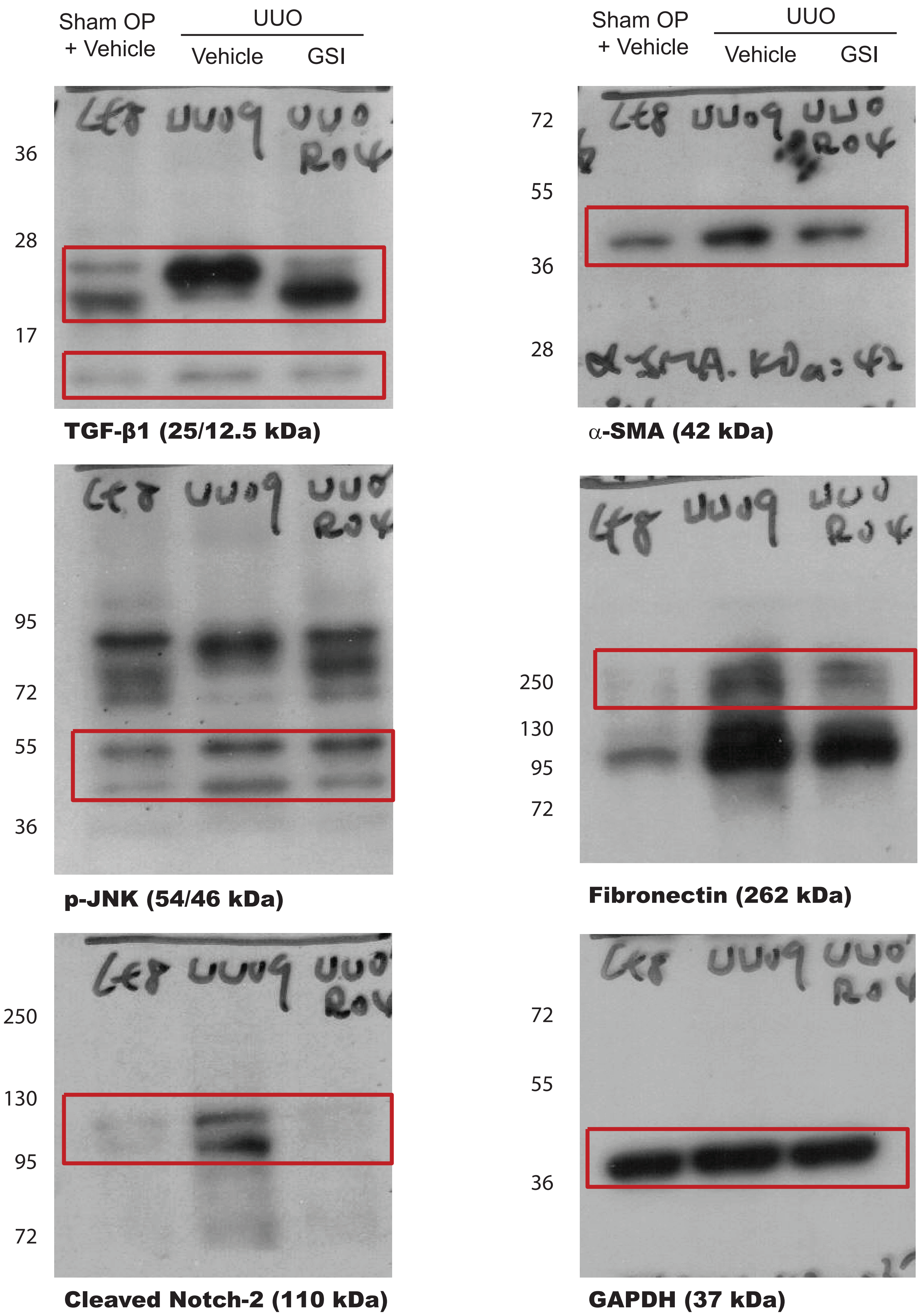

Figure S11, Full Length Western-blot images for Fig. 6b. (GSI: γ-secretase inhibitor, RO4929097)

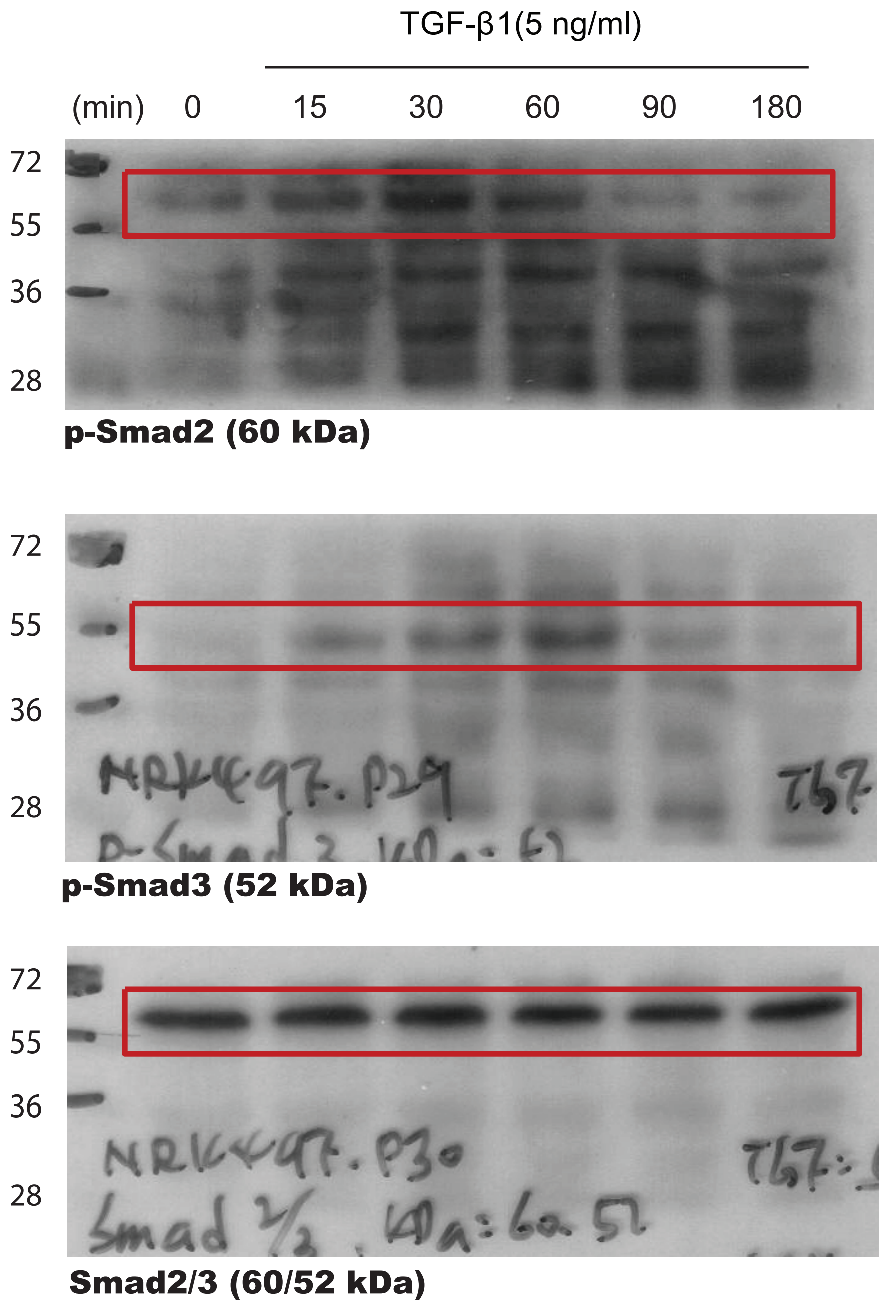

Figure S12, Full Length Western-blot images for Fig. S1c.

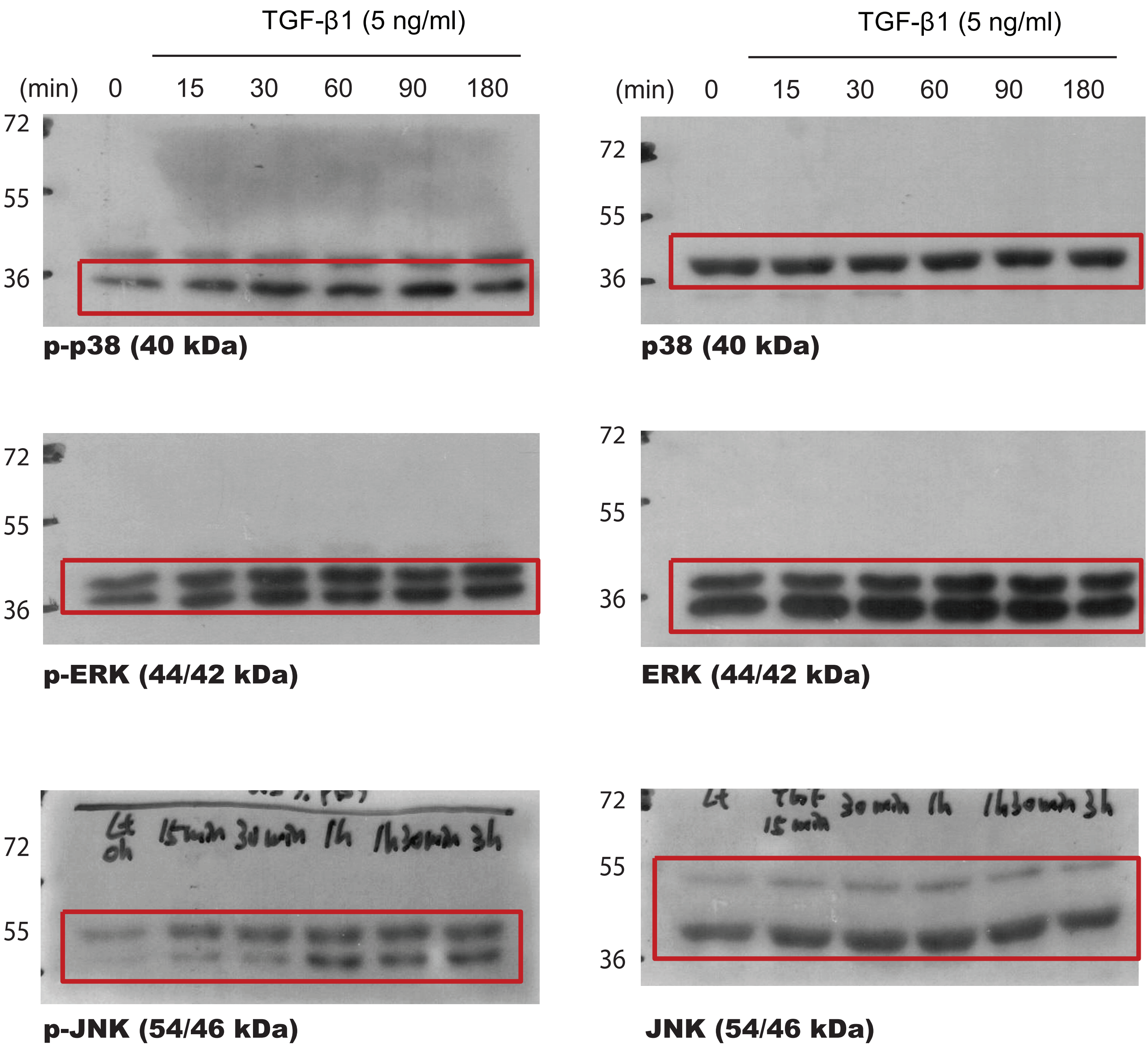

Figure S13, Full Length Western-blot images for Fig. S1d.

Fig. S2a

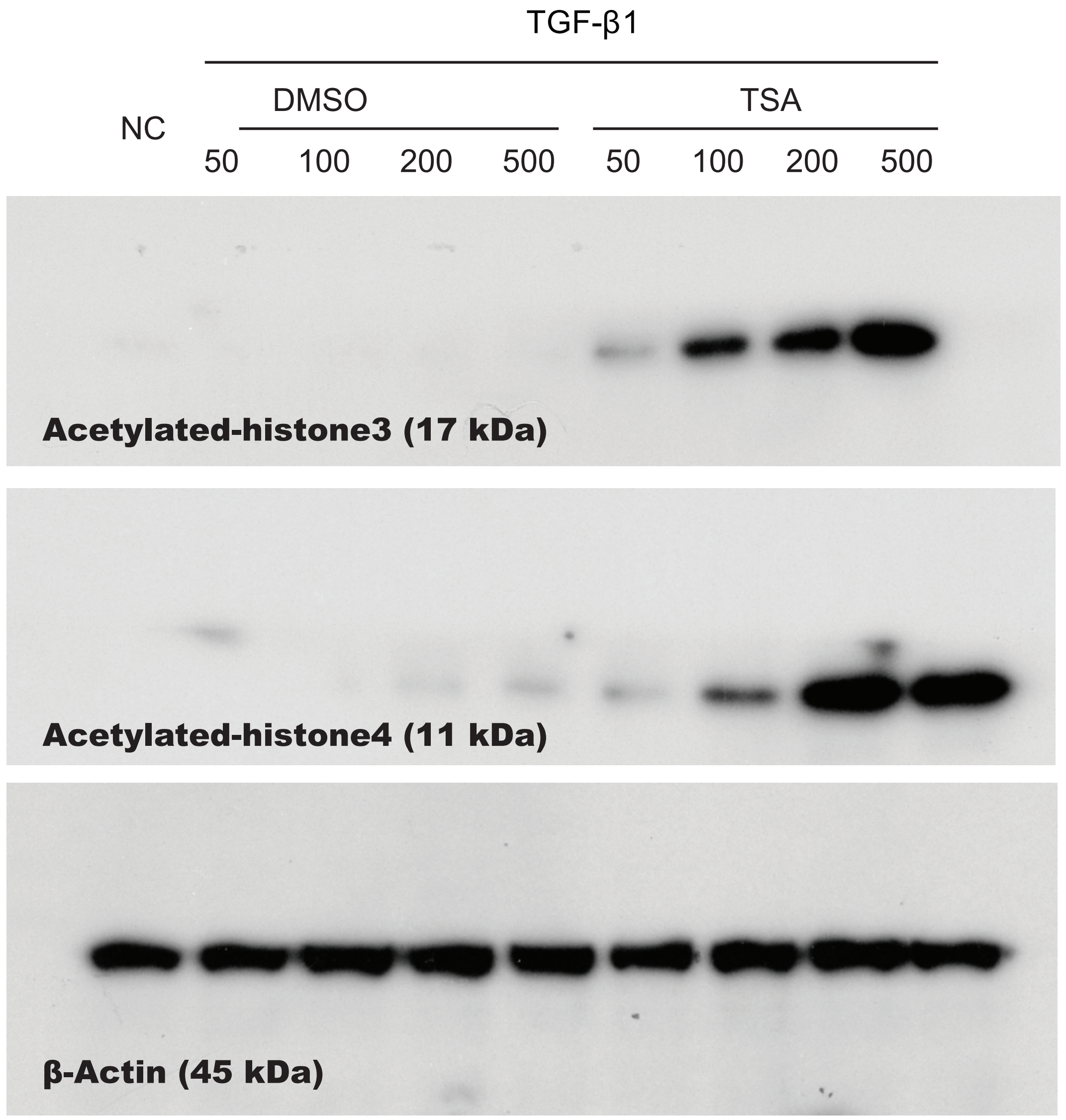

Fig. S2b

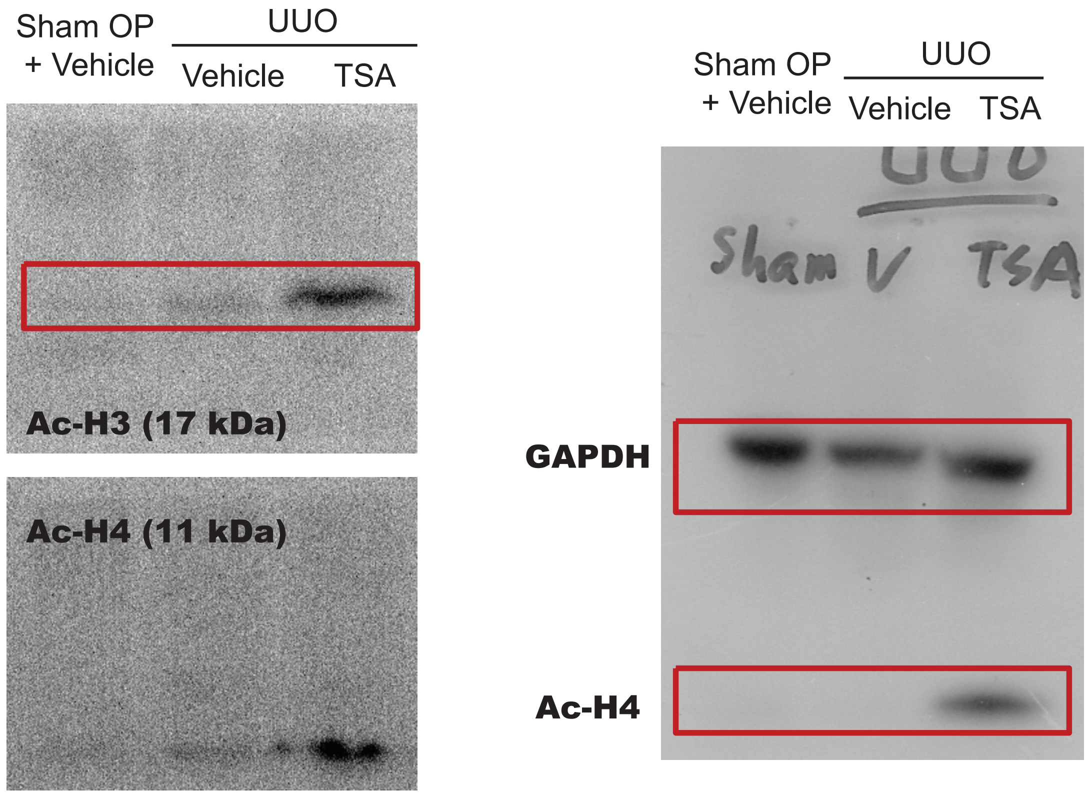

Figure S14, Full Length Western-blot images for Fig. S2.

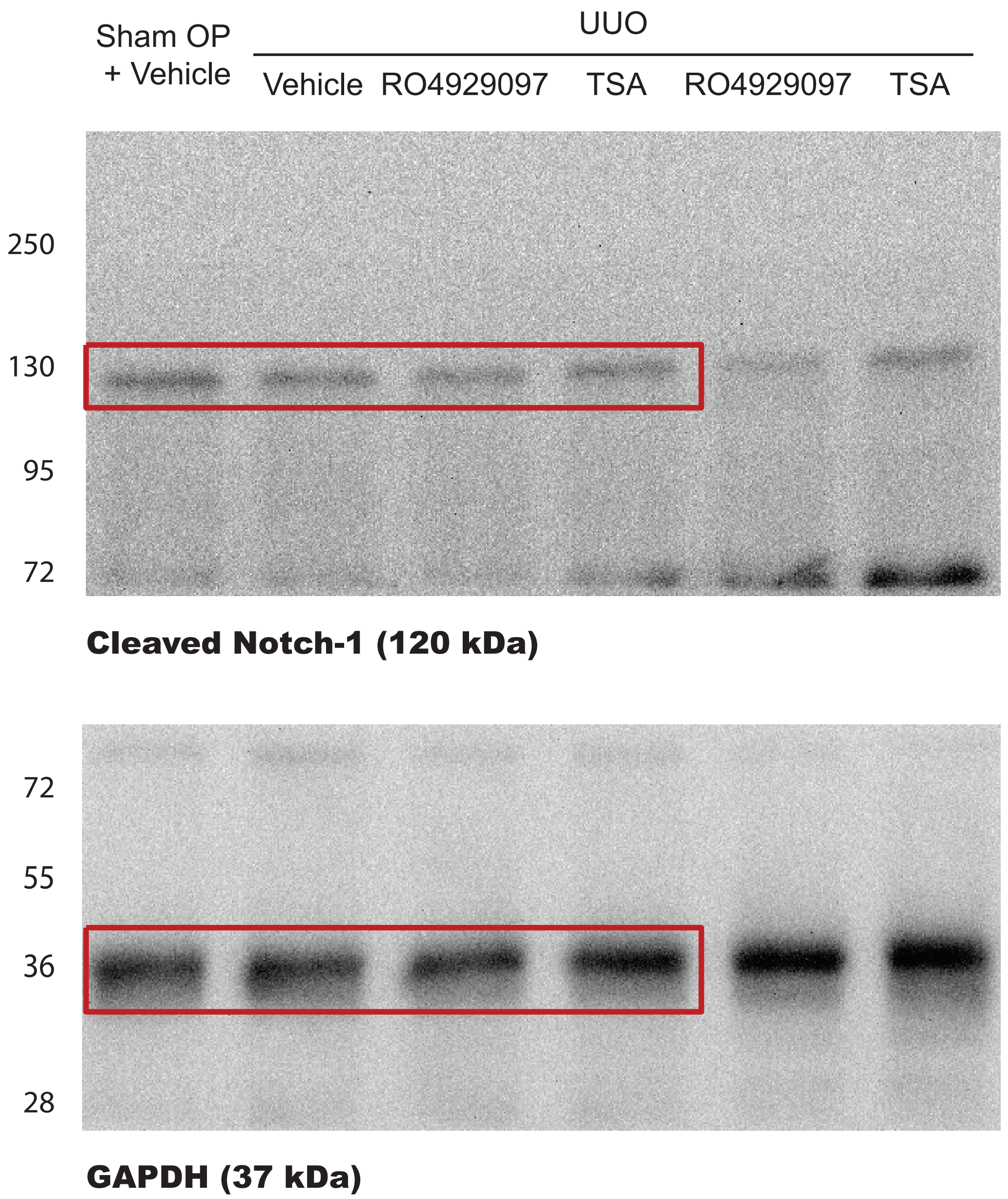

Figure S15, Full Length Western-blot images for Fig. S3.
